# A dual-task deep learning framework for automated detection and classification of coronary artery lesions in invasive coronary angiography imaging

**DOI:** 10.1186/s40001-025-03818-3

**Published:** 2026-01-30

**Authors:** Sha Ren Gao Wa, Hairong Tian, Xiao Xiao, Ning Liu, Haiyan Tian

**Affiliations:** 1 Ultrasound Department, The Affliated Hospital of lnner Mongola Medical University, Hohhot, 010030 Inner Mongolia China; 2 Infectious Diseases Department, Alshaa League Central Hospital, Bayanhot, 750306 Inner Mongolia China; 3 Interventional Therapy Department, Peking University Cancer Hospital (Inner Mongolia), Hohhot, 010020 Inner Mongolia China

**Keywords:** Coronary lesion detection, Coronary lesion classification, Deep learning, Dual-task framework

## Abstract

**Objective:**

This study aims to develop and evaluate a dual-task deep learning framework for the simultaneous detection and classification of coronary lesions in invasive coronary angiography (ICA).

**Materials and methods:**

A retrospective analysis was conducted using an annotated ICA dataset comprising 1234 patients (14,808 lesion-positive and 11,872 lesion-negative images), along with an external dataset of 135 cases. ICA video sequences were converted into 512 × 512 resolution images. A comprehensive preprocessing pipeline, including intensity normalization, noise suppression, and diverse data augmentation techniques (e.g., rotation, flipping, scaling, and brightness/contrast tuning), was applied to standardize image quality and enhance model generalizability. The proposed framework incorporates a detection module (utilizing YOLOv11, Swin Transformer, DETR, and Deformable DETR) and a classification module (featuring Vision Transformer (ViT), Swin Transformer, and ConvNeXt). The data were partitioned into training, validation, and testing subsets in an 80:10:10 ratio, and hyperparameter tuning was performed via grid search. Detection loss functions included Intersection over Union (IoU)-based losses, L1, and GIoU, while classification relied on weighted binary cross-entropy. Performance was evaluated using IoU, mAP, sensitivity, specificity, and AUC-PR.

**Results:**

The detection module exhibited consistent high performance across both internal and external datasets. Deformable DETR achieved superior results on the external dataset, with a mAP of 88.2%, IoU of 87.0%, sensitivity of 92.0%, and specificity of 90.1%, outperforming other models. For classification, the Swin Transformer reached an external accuracy of 92.8%, AUC-PR of 0.94, and a Cohen’s Kappa of approximately 0.89, exceeding the performance of ConvNeXt and ViT. These results are comparable to human expert performance in coronary lesion detection, where accuracy ranges from 85 to 90%, demonstrating the system’s robust diagnostic capability. The achieved sensitivity and specificity values are clinically meaningful, as they reduce the risk of both false positives and false negatives, crucial for ensuring timely and accurate treatment decisions in clinical practice.

**Conclusion:**

The proposed dual-task deep learning framework demonstrates a significant advancement in automated coronary lesion assessment by enabling accurate and efficient simultaneous detection and classification, thus supporting enhanced clinical decision-making.

**Supplementary Information:**

The online version contains supplementary material available at 10.1186/s40001-025-03818-3.

## Introduction

Coronary artery disease is a leading cause of mortality worldwide, making early and accurate diagnosis critical for effective treatment and management [1, 2]. Invasive coronary angiography (ICA) is the clinical gold standard for visualizing coronary arteries and assessing stenotic lesions, providing dynamic image sequences suitable for automated lesion analysis [3, 4] . However, the manual interpretation of lesions is often time-consuming and prone to inter-observer variability. These challenges have spurred significant interest in developing automated approaches that can reliably detect and classify coronary artery lesions [5, 6]. The recent advancements in deep learning have revolutionized computer vision, particularly in tasks, such as object detection and image classification [7, 8]. Transformer-based models, initially designed for natural language processing, have been successfully adapted for vision applications, demonstrating remarkable performance improvements [9, 10]. In this context, we propose a novel dual-task deep learning framework for the automated detection and classification of coronary artery lesions in ICA-derived imaging. Our framework integrates a suite of state-of-the-art models, each contributing unique strengths that address the inherent challenges of ICA image analysis.

The recent advances in deep learning have transformed medical image analysis, particularly in cardiovascular imaging, by enabling accurate and automated detection of subtle lesions. In developing the proposed dual-task framework, we strategically selected a combination of transformer-based and CNN-inspired models to address the distinctive challenges of ICA imaging, including high vascular motion, low contrast, and the small size of coronary lesions. Transformer-based architectures, such as Swin Transformer [11–13], Deformable DETR (Deformable DEtection TRansformer) [14], DETR [15], and YOLOv11 for lesion detection [16], along with Swin Transformer [17], Vision Transformer (ViT) [18], and ConvNeXt [19] were chosen for their capacity to model long-range spatial dependencies and capture multiscale contextual information, both essential for detecting fine vascular abnormalities within complex angiographic backgrounds. In particular, Deformable DETR was incorporated for its ability to focus attention adaptively on irregular and small anatomical regions, improving lesion localization accuracy in dynamic ICA sequences. For real-time lesion detection, YOLOv11 was integrated due to its computational efficiency and robust performance in scenarios demanding rapid analysis, such as intra-procedural coronary assessments [20].

For classification, ViT, Swin Transformer, and ConvNeXt provide complementary strengths. ViT leverages transformer mechanisms by dividing images into small patches, achieving competitive performance on large datasets like ImageNet. ConvNeXt enhances classical CNN-based architectures with insights from modern transformer models [21], achieving high classification accuracy by improving efficiency and model optimization. The combination of these models ensures comprehensive coverage of both global vessel context and localized lesion characteristics, enhancing the framework’s ability to generalize across diverse patient cases and imaging conditions. This strategic integration of architectures was therefore guided not by architectural novelty alone but by the practical clinical objective of achieving accurate, efficient, and interpretable automated lesion assessment in invasive coronary angiography.

The recent advances in deep learning have significantly enhanced the automated analysis of cardiovascular imaging, with several studies demonstrating notable progress in IVUS and CT angiography applications. Szarski and Chauhan [22] developed a coordinate-aware fully convolutional network that leverages polar translation dependence to achieve real-time segmentation of IVUS images, accurately delineating lumen and media with minimal computational cost and outperforming traditional methods. In a complementary approach, Nishi et al. [23] introduced a deep learning-based framework for IVUS segmentation that not only segments the lumen and vessel areas but also extends to stent region delineation, achieving excellent agreement with expert annotations across extensive datasets. Li and et al. [24] further contributed by designing an end-to-end convolutional neural network that integrates cascaded U-Nets and evaluates multiple loss functions, thereby enabling robust detection of media–adventitia borders, luminal regions, and calcified plaques, even in the presence of imaging artifacts. Extending these efforts to coronary CT angiography, Zreik et al. [25] proposed a recurrent CNN that combines 3D convolutional feature extraction with sequential processing, effectively detecting and classifying different types of coronary artery plaque and stenosis with promising accuracy. In a subsequent study [26], the same group applied deep learning to analyze left ventricle myocardium in CT angiography, employing multiscale segmentation and unsupervised feature encoding to identify patients with functionally significant coronary stenosis, potentially reducing the need for invasive fractional flow reserve measurements. In addition, Alizadehsani et al. [27] presented a novel machine learning algorithm that uses dedicated classifiers for stenosis prediction in the LAD, LCX, and RCA, achieving high accuracy, sensitivity, and specificity for coronary artery disease detection, thereby offering a noninvasive alternative to traditional angiography. Collectively, these contributions underscore a concerted effort to harness advanced deep learning architectures for improving diagnostic precision, computational efficiency, and clinical applicability in cardiovascular imaging.

Although dual-task or multitask deep learning strategies have previously been explored in related cardiovascular imaging modalities such as IVUS and coronary CT angiography (CCTA), these approaches are typically designed for segmentation–classification or plaque characterization tasks within static or quasi-static imaging settings. In contrast, ICA presents distinct technical challenges, including dynamic contrast propagation, projection-dependent vessel overlaps, rapid cardiac motion, and the presence of small, low-contrast lesions.

The novelty of the present work therefore does not lie in proposing dual-task learning in a generic sense, but in developing a comprehensive, ICA-specific dual-task framework that integrates state-of-the-art transformer-based detection models with complementary classification architectures in a unified and clinically aligned pipeline. To the best of our knowledge, no prior study has systematically evaluated and integrated deformable and hierarchical transformer-based detectors with transformer- and CNN-inspired classifiers for simultaneous coronary lesion detection and classification directly from ICA images, with validation on an independent external cohort. This modality-specific design and evaluation constitute the primary contribution of the proposed framework. The key innovations of our study are summarized as follows:

1. The development of the first comprehensive dual-task deep learning framework that concurrently addresses both detection and classification of coronary artery lesions in ICA imaging.

2. The application of hierarchical and deformable transformer-based architectures to enhance detection performance and streamline computational efficiency.

3. The strategic integration of transformer-based and CNN-inspired models to achieve superior classification accuracy for a wide range of lesion types.

4. A thorough evaluation and comparison of multiple model configurations to identify the optimal balance between real-time processing and high-resolution medical image analysis.

**Fig. 1:**
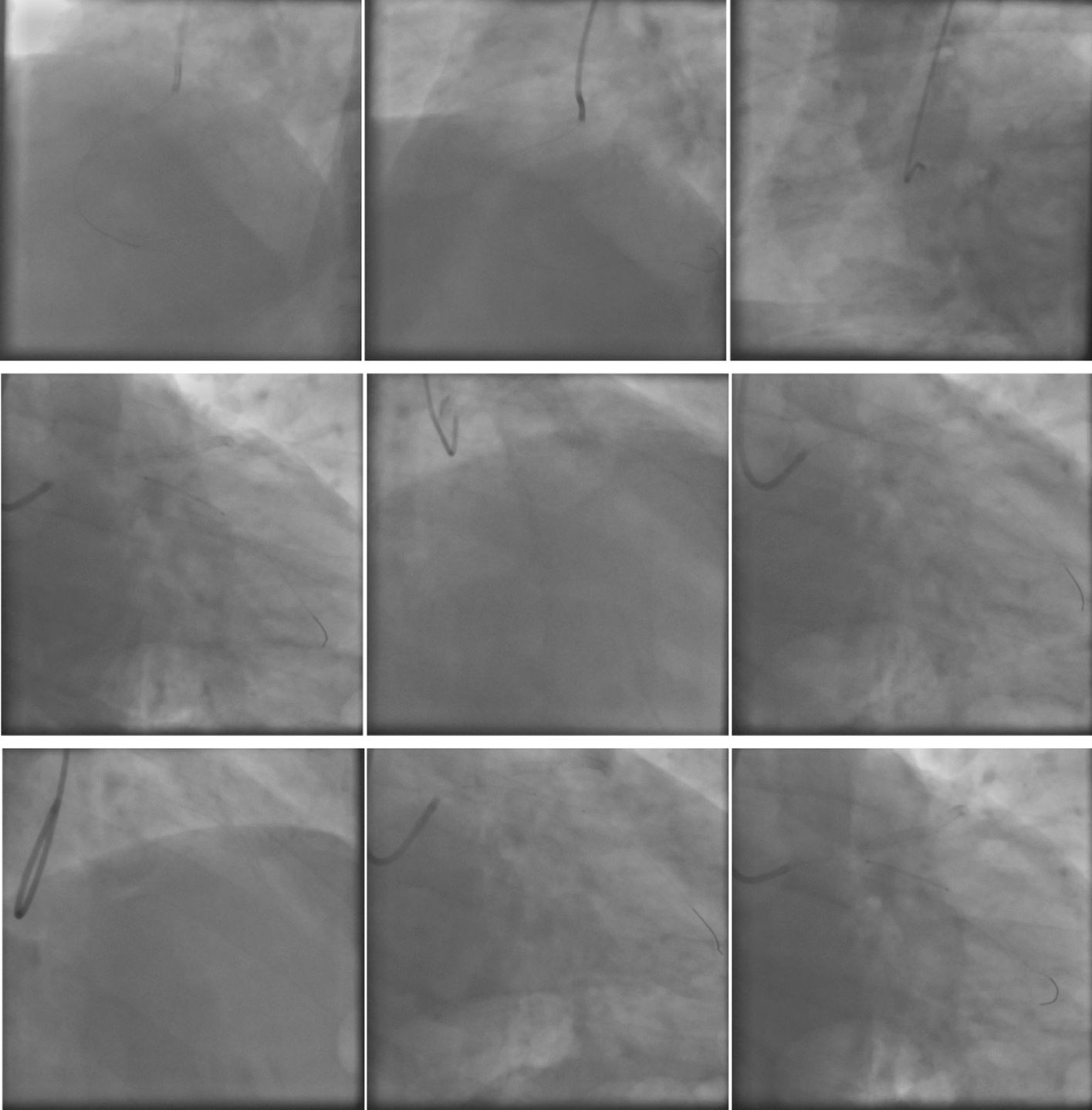
Examples of coronary artery lesions in ICA images

In total, the retrospective dataset comprised 1234 patients (primary cohort) and an external validation set of 135 independent cases. The patient population included individuals presenting with both stable angina (approximately 44%) and acute coronary syndrome (approximately 34%), representing different stages of coronary artery disease progression. Follow-up clinical assessments indicated that the majority of patients who underwent percutaneous coronary intervention (PCI) or received optimized medical management exhibited notable symptomatic recovery and improved left ventricular function during subsequent evaluations. These data provide a representative clinical foundation for developing and validating the proposed automated diagnostic framework. These innovations not only set a new benchmark in AI-driven cardiovascular diagnostics but also provide a scalable and efficient solution for coronary artery lesion assessment, paving the way for more reliable and timely clinical decision-making. Figure 1 shows examples of coronary artery lesions in ICA images. These lesions are the focus of the proposed deep learning framework for detection and classification.

## Materials and methods

### Datasets

#### Data acquisition

The primary dataset consists of an annotated Invasive Coronary Angiography (ICA) collection from 1234 patients, retrospectively acquired between 2015 and 2023. The ICA examinations were performed using the Artis Zee system (Siemens AG, Munich, Germany) under standardized clinical protocols. The clinical dataset for this study was collected over five years from two tertiary care hospitals and an affiliated diagnostic center. Ethical approval for this study was waived by the Ethical Committee of Inner Mongolia Medical University, Affiliated Hospital, Inner Mongolia, 010030, China. Informed consent was obtained from all participants involved in the study. For individuals under the age of 18, written consent was secured from their parents or legal guardians. All participants were thoroughly informed about the study’s objectives, procedures, and their right to withdraw at any time without consequences. In addition, all experiments were conducted in compliance with relevant ethical guidelines and regulations, and all methodologies adhered to the applicable guidelines and regulations outlined in the manuscript.

For the left coronary artery (LCA), five projections were typically recorded, including right anterior oblique (RAO) and left anterior oblique (LAO) views with both cranial and caudal angulations, with additional views obtained when diagnostic ambiguity arose. For the right coronary artery (RCA), the standard RAO and LAO projections with cranial and caudal angulations were employed. The angiography videos were captured in DICOM format at a rate of 10 frames per second, with each acquisition lasting between 4 and 8 s depending on the projection. Consequently, the video sequences varied in length, ranging from a single frame to 170 frames per study. For streamlined management and processing, these videos were converted to PNG images at a resolution of 512 × 512 pixels. In addition, an external dataset comprising 135 cases was incorporated to further validate the robustness and generalizability of the proposed framework.

All ICA frames were standardized to a resolution of 512 × 512 pixels prior to model training. This resolution was selected to match the native spatial scale of the angiography acquisition system while ensuring consistent input dimensions across patients, projections, and imaging protocols. Importantly, resizing was performed using aspect-ratio-preserving interpolation to avoid geometric distortion of coronary vessels and lesions. The chosen resolution provides an effective balance between preserving fine vascular morphology, such as luminal narrowing and edge irregularities, and maintaining computational efficiency and training stability, particularly for transformer-based detection architectures. Empirically, 512 × 512 resolution was found sufficient to retain clinically relevant lesion features while enabling scalable training and robust generalization across datasets.

Although invasive coronary angiography is inherently dynamic, the conversion of ICA videos into frame-based PNG images was intentionally adopted in this study to align with clinical interpretation practices and annotation feasibility. In routine catheterization laboratory workflows, lesion assessment is primarily based on selected frames that provide optimal contrast filling and projection angles, rather than continuous temporal analysis. Accordingly, frames with clearly visible coronary lesions were identified and annotated by expert cardiologists, ensuring that the extracted images captured clinically meaningful lesion characteristics. This frame-based strategy enables precise spatial annotation, scalable dataset construction, and robust training of detection and classification models, while maintaining fidelity to real-world diagnostic decision-making.

The clinical and demographic characteristics of the patients from both the primary and external datasets are summarized in Table 1*.* The table includes key features such as age, sex, comorbid conditions, angiography indications, and coronary artery involvement. These attributes provide an overview of the study population, ensuring a comprehensive understanding of the datasets used for training and validating the proposed deep learning framework.

**Table 1:** Clinical and demographic characteristics of the patients in the study

Characteristic	Primary dataset (*n* = 1234)	External dataset (*n* = 135)
Age (years**)**	Mean ± SD: 65.3 ± 10.7	Mean ± SD: 66.1 ± 9.5
Sex:	Male: 814 (66.0%)	Male: 86 (63.7%)
	Female: 420 (34.0%)	Female: 49 (36.3%)
Body Mass index (BMI, kg/m^2^)	Mean ± SD: 27.8 ± 4.1	Mean ± SD: 28.1 ± 4.4
Hypertension	Yes: 782 (63.4%)	Yes: 85 (63.0%)
Diabetes mellitus	Yes: 452 (36.6%)	Yes: 48 (35.6%)
Hyperlipidemia	Yes: 621 (50.3%)	Yes: 67 (49.6%)
Smoking history: Current Former Never		
	315 (25.5%)	34 (25.2%)
	254 (20.6%)	28 (20.7%)
	665 (53.9%)	73 (54.1%)
Family history of CAD	Yes**:** 492 (39.9%)	Yes**:** 49 (36.3%)
Previous myocardial infarction	Yes**:** 276 (22.4%)	Yes**:** 29 (21.5%)
Previous percutaneous coronary intervention (PCI)	Yes**:** 195 (15.8%)	Yes**:** 22 (16.3%)
Previous coronary artery bypass grafting (CABG)	Yes**:** 114 (9.2%)	Yes**:** 12 (8.9%)
Left ventricular ejection fraction (LVEF)	Mean ± SD**:** 53.1 ± 8.9%	Mean ± SD**:** 52.8 ± 9.3%
Angiography indication		
Stable angina	542 (44.0%)	58 (43.0%)
Acute coronary syndrome	418 (33.9%)	44 (32.6%)
Other	274 (22.2%)	33 (24.4%)
Coronary artery involvement		
Single-vessel disease	398 (32.3%)	45 (33.3%)
Multi-vessel disease	836 (67.7%)	90 (66.7%)
Stenosis severity		
Mild (≤ 50%)	382 (31.0%)	43 (31.9%)
Moderate (50–70%)	524 (42.5%)	60 (44.4%)
Severe (> 70%)	328 (26.6%)	32 (23.7%)
Medications at Time of Angiography		
Aspirin	Yes: 1,019 (82.6%)	Yes: 112 (83.0%)
Statins	Yes: 968 (78.4%)	Yes: 106 (78.5%)
Beta-blockers	Yes: 574 (46.5%)	Yes: 58 (43.0%)
ACE Inhibitors/ARBs	Yes: 612 (49.6%)	Yes: 66 (48.9%)
Imaging duration (s)	Median (IQR): 6.3 (4.5–8.1)	Median (IQR): 6.1 (4.2–7.9)
Frames per study	Mean ± SD: 103 ± 35	Mean ± SD: 101 ± 32

**Fig. 2:**
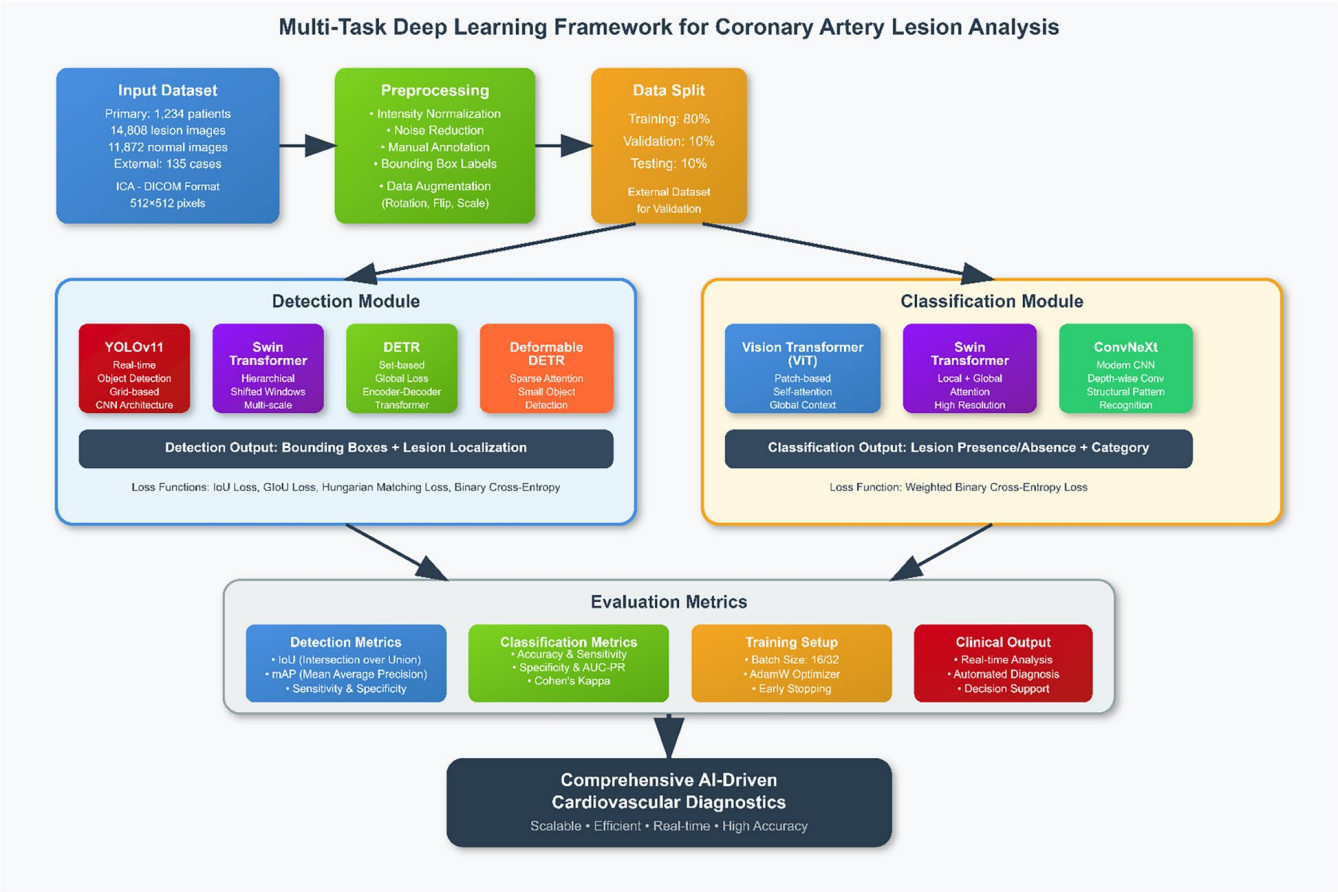
Dual-task deep learning framework for coronary artery lesion detection and classification

Figure 2 illustrates the proposed dual-task deep learning framework designed for the simultaneous detection and classification of coronary artery lesions in ICA images. The framework consists of a detection module (YOLOv11, Swin Transformer, DETR, and Deformable DETR) and a classification module (ViT, Swin Transformer, and ConvNeXt). The dataset is split into training, validation, and test sets, with an external dataset used for independent evaluation.

#### Inclusion and exclusion criteria

The inclusion and exclusion criteria were established to ensure that high-quality, clinically relevant data were used for both detection and classification tasks. The criteria for each task are detailed below:

#### Detection task

### Inclusion criteria

1. Images containing clearly visible coronary artery lesions.

2. Frames where lesions were fully within the field of view.

3. Annotated frames with high-resolution and sufficient contrast for bounding box delineation.

4. Angiography frames from projections that followed standardized clinical protocols.

#### Exclusion criteria

1. Frames with severe imaging artifacts (e.g., motion blur, noise, or occlusions).

2. Images where lesions were partially visible or ambiguous in appearance.

3. Frames with incomplete clinical data (e.g., missing metadata or corrupted images).

4. Noninformative frames that lacked any relevant coronary structures.

#### Classification task

### Inclusion criteria

1. Images labeled with clear lesion presence (positive cases) or absence (negative cases).

2. Frames with standardized resolution (512 × 512 pixels) and acceptable image quality.

3. Cases with full diagnostic imaging data, including projections from all relevant coronary arteries.

### Exclusion criteria

1. Images with low signal-to-noise ratios or degraded resolution.

2. Frames exhibiting unclear or ambiguous coronary anatomy.

3. Cases where critical clinical data (e.g., lesion type, location, or classification) were unavailable.

4. Frames with preprocessing errors, including failed normalization or alignment issues.

These criteria ensured that both detection and classification tasks were trained on robust, high-quality data, minimizing the risk of poor model generalization due to noisy or incomplete inputs.

### Data preprocessing

Following conversion to PNG format, all images underwent preprocessing to standardize their quality and format. This included intensity normalization to ensure uniform contrast across the dataset and noise reduction to mitigate imaging artifacts.

Noise suppression was performed using a two-stage denoising strategy to reduce imaging artifacts although preserving fine vascular structures. First, a mild Gaussian filter (kernel size 3 × 3, σ = 0.8) was applied to attenuate high-frequency electronic noise commonly present in ICA acquisitions. Subsequently, nonlocal means (NLM) filtering was employed to further suppress stochastic noise while maintaining vessel edges and lesion boundaries. For NLM, the search window size was set to 21 × 21 pixels, the patch size to 7 × 7 pixels, and the filtering strength parameter (h) was empirically tuned on the training set to minimize visual blurring of coronary contours. Parameter selection was guided by qualitative expert inspection and quantitative stability of downstream detection performance, ensuring that denoising improved image quality without distorting clinically relevant vessel morphology.

A detailed manual annotation process was performed, whereby frames containing visible coronary artery lesions were identified and marked with bounding boxes. Given that each angiography video comprised between 1 and 170 frames, with lesions appearing intermittently, only frames with clearly visible lesions were annotated. The dataset was thus divided into two categories:

Images with visible lesions: A total of 14,808 images, each with corresponding bounding box annotations.

Images without visible lesions: A total of 11,872 images labeled as having no detectable lesions.

Noninformative or corrupted frames were excluded from the dataset. For consistency, all image sequences were standardized in length through padding or temporal cropping techniques to ensure uniform input dimensions for the deep learning models. This preprocessing pipeline aimed to maximize the effectiveness of both the detection and classification tasks.

#### Annotation process and inter-observer agreement

All lesion annotations were performed by three independent experts to ensure accuracy and reproducibility. The annotation team consisted of two senior interventional cardiologists (each with over ten years of clinical experience in ICA interpretation) and one imaging research scientist with expertise in computer vision and medical image processing. Bounding box annotations for lesion detection were generated using the LabelImg software, while lesion classification labels (calcified vs. noncalcified) were assigned based on established angiographic appearance criteria. To assess annotation consistency, inter-observer agreement was quantified using Cohen’s Kappa for classification (κ = 0.88) and mean Intersection over Union (IoU = 0.86) for detection bounding boxes, both reflecting excellent reliabilities. Any disagreements were reviewed collaboratively and resolved by consensus to produce a unified ground truth dataset.

#### Data augmentation

**Fig. 3:**
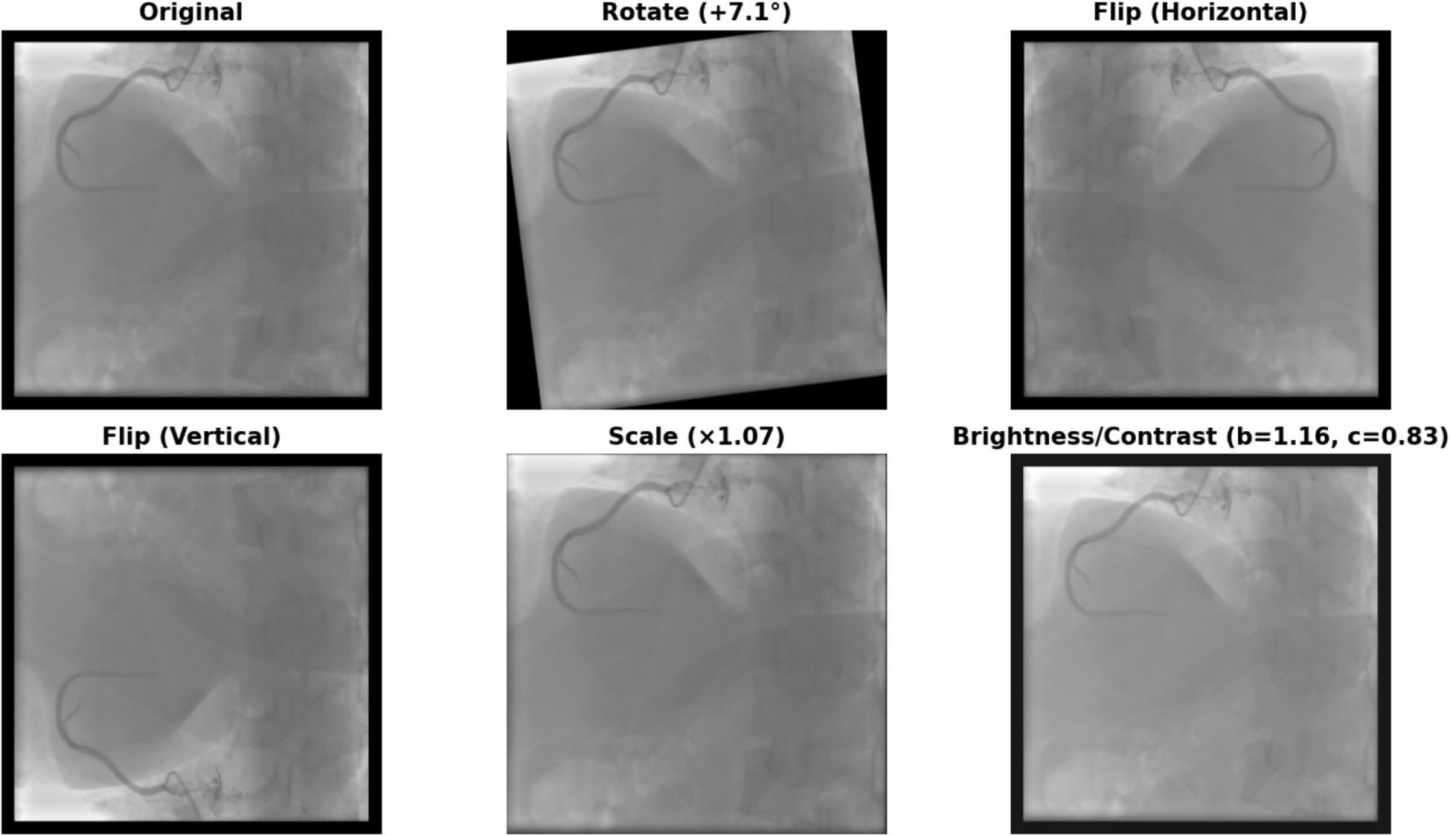
Representative examples of data augmentation applied to ICA images

To enhance model robustness and minimize the risk of overfitting, a comprehensive set of data augmentation techniques was employed [28]. These augmentations were designed to introduce variability into the training data, simulating real-world conditions that may arise in ICA imaging (Fig. 3). First, random rotations of up to ± 15 degrees were applied, ensuring that the model could handle differences in image orientation. In addition, horizontal and vertical flipping were incorporated to account for variations in anatomical positioning during imaging. Scaling transformations, ranging from a factor of 0.8 to 1.2, were utilized to simulate changes in image size. Brightness and contrast adjustments were also introduced to reflect the variability in lighting and contrast settings across different imaging devices and patient conditions. These augmentations were carefully parameterized to prevent unrealistic alterations while effectively capturing clinically relevant variations. The data augmentation techniques, such as random rotations, flips, scaling, and brightness adjustments were applied to both the detection and classification modules. For the detection module, bounding box annotations were adjusted dynamically to match the augmented images.

### Dual-task framework overview

Although the proposed framework includes both a detection module and a classification module, it is more accurately described as a dual-task system rather than a dual-task model. In this system, the detection module first identifies and localizes lesions within the ICA images, and the classification module subsequently categorizes these lesions based on the detected regions. This approach enables end-to-end lesion analysis, where the outputs of the detection module directly inform the classification process, improving overall model performance in a cohesive and integrated manner. The framework integrates multiple deep learning models, each optimized for either detection or classification tasks. The detection module leverages both transformer-based models and a CNN-based architecture (YOLOv11), while the classification module incorporates ViT, Swin Transformer, and ConvNeXt. This architecture was developed to balance computational efficiency with high detection accuracy across varying image conditions, enabling real-time and scalable analysis of coronary artery lesions.

The dual-task framework is composed of two primary components: the detection module and the classification module. Input images, after preprocessing and augmentation, are fed into the detection module, which identifies and localizes lesions using bounding box annotations. The frames are then passed to the classification module, which determines whether a lesion is present and, if so, its category (e.g., calcified or noncalcified). Each component leverages multiple models with complementary strengths to improve performance across a range of lesion types and image quality variations. The detection module employs both YOLOv11 and transformer-based architectures (Swin Transformer, DETR, and Deformable DETR), while the classification models are designed to generalize across both positive and negative cases with minimal inter-observer variability.

#### Detection and classification modules

**Fig. 4:**
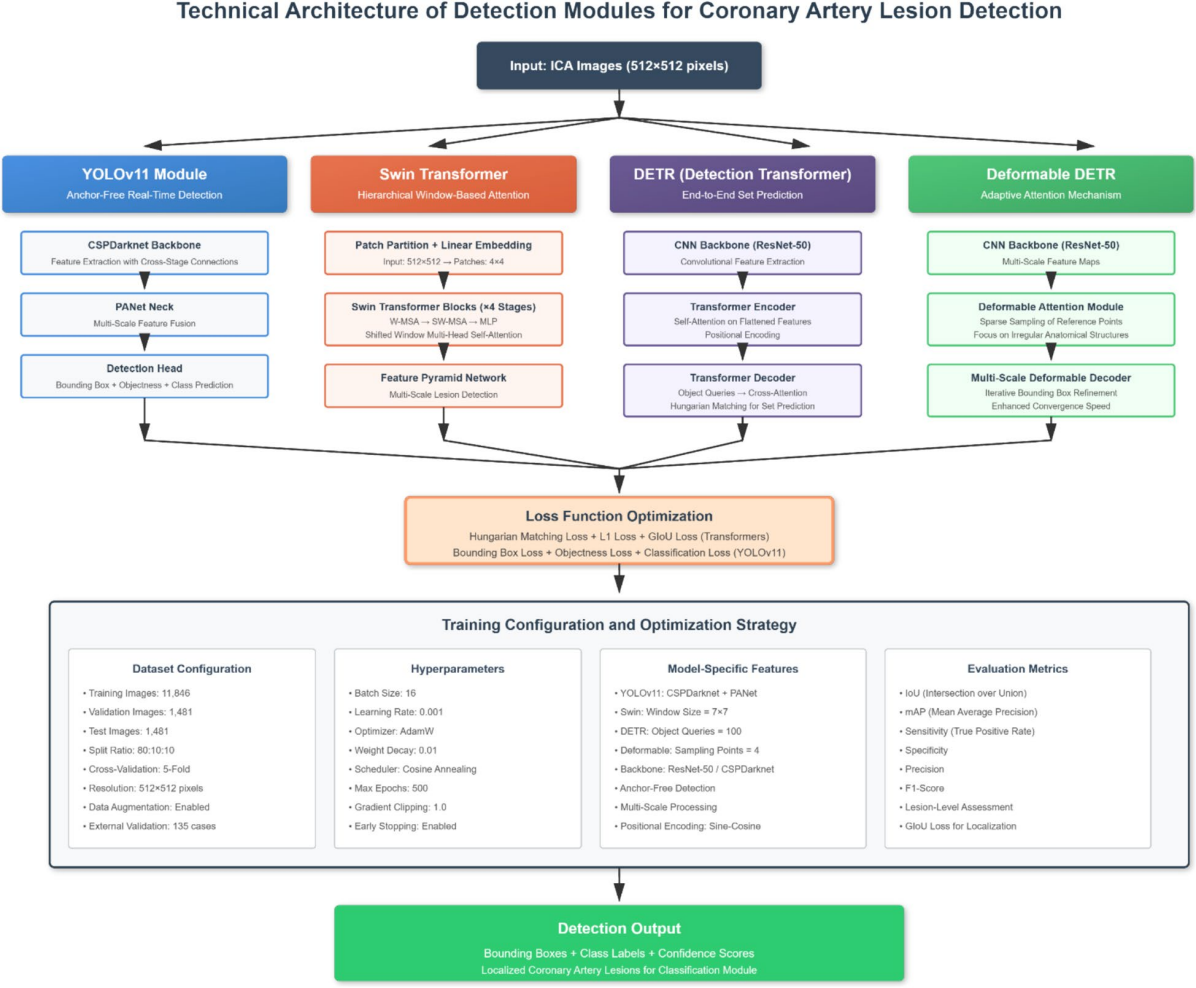
Block diagram of the detection module architectures

**Fig. 5:**
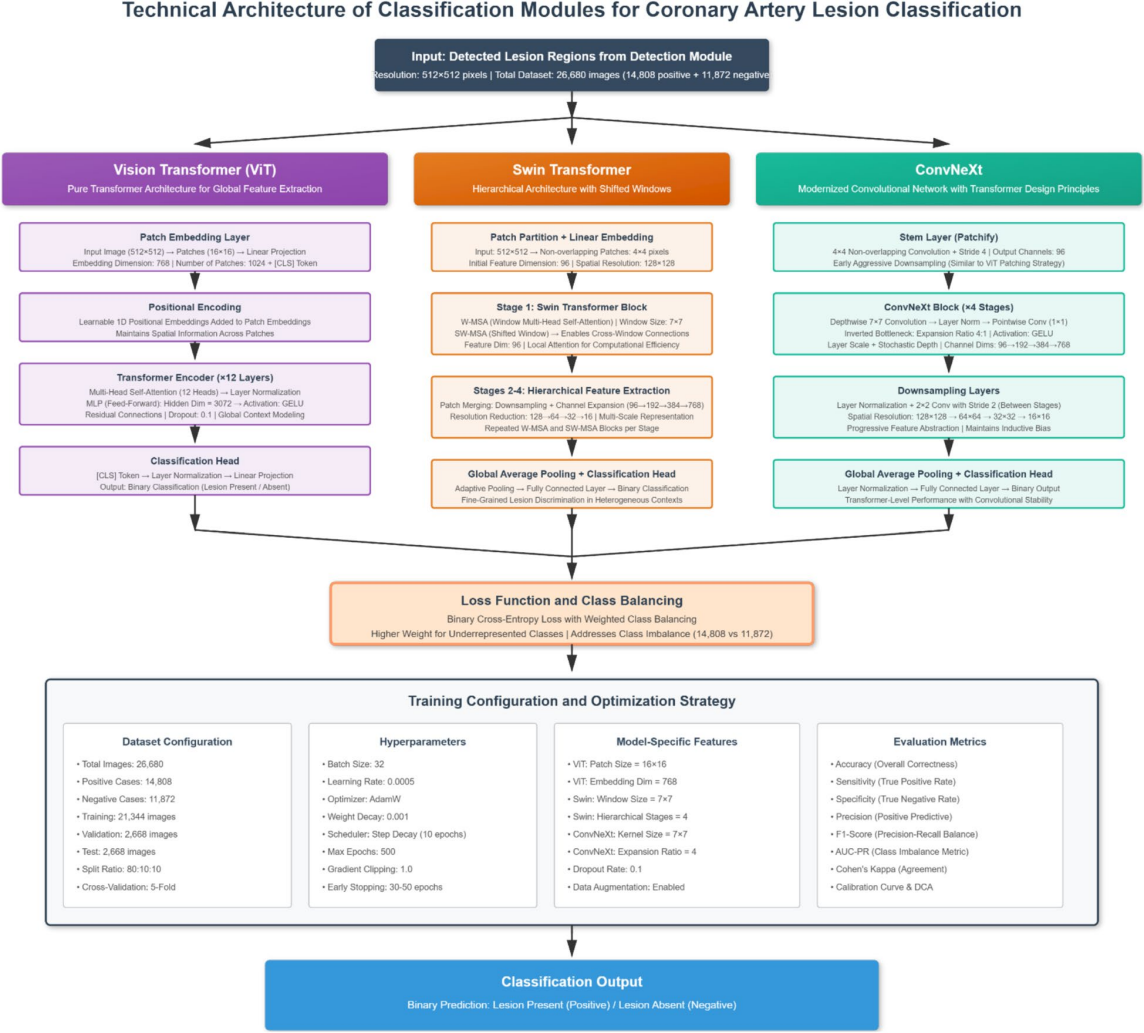
Block diagram of the classification module architectures

The proposed dual-task framework contains complementary architectures to combine their different merits in both detection and classification tasks (Figs. 4 and 5). For lesion detection, the models of YOLOv11, Swin Transformer, DETR, and Deformable DETR were used. YOLOv11 was chosen as a high-speed and anchor-free baseline model, providing real-time detection capability with decent localization performance for dynamic ICA sequences. The Swin Transformer increases the detection accuracy by using hierarchical window-based attention, which can achieve better multiscale representation and lesion size robustness. DETR (Detection Transformer) proposes an end-to-end transformer-based detection mechanism that eliminates the need for anchor generation and post-processing, which improves the interpretability of the detection mechanism while reducing the architectural complexity. Deformable DETR, an advanced extension of DETR, was selected, which has adaptive attention mechanism focusing on irregular and small anatomical structures which would improve convergence speed and localization accuracy in complex vascular images.

ViT, Swin Transformer and ConvNeXt were used for lesion classification. ViT provides a purely transformer-based global feature extraction architecture that is able to capture subtle visual cues over the whole angiographic frame. Swin Transformer with shifted window architecture provides efficient hierarchical representation and is good at fine grained lesion discrimination within heterogeneous image context. ConvNeXt is a next-generation convolutional network, which is chosen to offer a strong convolutional baseline at transformer level performance, to improve stability and generalization. The combination of these models makes it possible to achieve complementary learning between convolutional and transformer-based paradigms, both in terms of high detection sensitivity and high classification reliability for various types of coronary lesions.

The use of lesion-negative frames differs between the detection and classification tasks by design and reflects the distinct learning objectives of each module. The detection task is formulated as a lesion localization problem and therefore requires only lesion-positive frames with bounding box annotations, as lesion-negative images do not provide spatial supervision for bounding box regression. Including a large proportion of lesion-negative frames in detection training may bias the model toward background prediction and reduce sensitivity to small or subtle coronary lesions, which are clinically critical in invasive coronary angiography.

In contrast, the classification task explicitly aims to discriminate between lesion-present and lesion-absent frames and therefore incorporates both lesion-positive (14,808 images) and lesion-negative (11,872 images) samples to learn a robust decision boundary. This asymmetry does not bias the dual-task framework, as the detection and classification modules are trained independently with task-specific objectives and loss functions. During inference, the detection module first localizes candidate lesions, and the classification module subsequently evaluates these regions of interest, ensuring complementary rather than conflicting learning behavior. This task–aware data utilization strategy prioritizes high detection sensitivity while maintaining balanced and clinically meaningful classification performance.

### Implementation details

The training process for both the detection and classification modules was carefully designed to optimize model performance while ensuring efficient convergence. The implementation leveraged both transformer-based and CNN-based architectures, with each model tuned to maximize detection accuracy and classification precision. The key aspects of the training procedure, hyperparameter settings, and loss functions are outlined below.

#### Training procedure

For both the detection and classification modules, the dataset was initially divided into training, validation, and test subsets in an 80:10:10 ratio to maintain balanced and unbiased evaluation. To further ensure comprehensive assessment and mitigate overfitting, a fivefold cross-validation procedure was implemented for both modules. In this approach, the dataset was randomly partitioned into five equal folds; in each iteration, four folds were used for training and onefold for validation. The process was repeated five times so that each subset served once as the validation fold. This cross-validation strategy enhanced the robustness, reliability, and generalization capability of the proposed framework across unseen data.

To prevent data leakage and artificially inflated performance, the fivefold cross-validation procedure was conducted at the patient level rather than the frame level. Specifically, all ICA frames extracted from a given patient were assigned exclusively to a single fold, ensuring that no patient contributed data to more than onefold across training and validation. This patient-wise stratification strategy is essential for ICA video-derived datasets, where frames from the same patient are highly correlated in anatomy, contrast dynamics, and acquisition geometry. By enforcing patient-level separation across folds, the reported cross-validation results more accurately reflect true generalization performance on unseen patients.

The dataset used for training comprised annotated ICA images collected from 1234 patients, with 14,808 images containing visible lesions and 11,872 images labeled as having no detectable lesions. Additionally, an external dataset with 135 cases was incorporated to further assess the robustness and generalizability of the framework, providing a total of 1620 images with visible lesions and 1330 images without visible lesions.

To avoid data leakage, the dataset partitioning was performed at the patient level rather than at the frame level. Specifically, each patient’s ICA study (including all extracted frames from all projections and time points) was assigned exclusively to a single subset (training, validation, or test), ensuring that no frames from the same patient appeared across multiple subsets. This design is essential for ICA video-derived datasets because adjacent frames within a study are highly correlated in anatomy, contrast dynamics, and acquisition geometry. The same patient-wise split strategy was applied consistently for both the detection and classification experiments. Furthermore, the external cohort (135 independent cases) was not used during training or model selection and was reserved solely for independent testing.

#### Detection task training

The detection module was trained on 14,808 lesion-positive images, each annotated with bounding boxes to indicate lesion locations. To ensure robust model evaluation and prevent overfitting, the dataset was split into training, validation, and test subsets with an 80:10:10 ratio. Specifically, 11,846 images were used for training, 1481 images for validation, and 1481 images for testing. The training data were augmented to enhance the model's ability to generalize under various clinical conditions. During the training, the images were resized to a resolution of 512 × 512 pixels, and a batch size of 16 was used. Each detection model (YOLOv11, Swin Transformer, DETR, and Deformable DETR) was trained to predict both bounding box coordinates and class labels. The training was conducted for up to 500 epochs, with early stopping applied based on the performance on the validation set. Regular validation during training ensured that the models maintained high accuracy without overfitting. The external dataset, containing lesion-positive and lesion-negative frames, was used for independent testing to evaluate the models' generalizability across different imaging environments. The models were fine-tuned based on the validation performance, focusing on small-object detection capabilities critical for lesion localization in angiography images.

#### Classification task training

The classification module was trained on the entire dataset, including 14,808 images with visible lesions and 11,872 images without lesions, resulting in a total of 26,680 images. The dataset was split into 21,344 images for training, 2668 images for validation, and 2668 images for testing. In the classification task, positive and negative lesion cases were balanced through weighted loss functions. The weights were adjusted based on the class frequencies to address the imbalance in lesion-positive and lesion-negative images during training and validation.

Data augmentation was applied to simulate real-world imaging variability. The classification models (Vision Transformer, Swin Transformer, and ConvNeXt) were trained to output binary predictions. Early stopping was employed after 30 to 50 epochs based on validation performance. The external dataset was used to further validate the classification models, ensuring they could generalize to new cases with varying lesion visibility and imaging conditions. This approach minimized false positives and false negatives, improving the framework’s diagnostic reliability.

The dataset was split into training, validation, and test subsets using an 80:10:10 ratio for both the detection and classification tasks. For detection, the model was trained exclusively on 14,808 lesion-positive images, while for classification, the model used the entire dataset of 26,680 images, including both lesion-positive and lesion-negative images. The training subsets for both tasks were mutually exclusive, ensuring no overlap of lesion-positive images between the two tasks. Details of these splits are provided in Table 2. The external dataset of 135 cases was used solely for independent testing.

**Table 2:** Dataset distribution for detection and classification tasks

Task / Dataset	Total images	Lesion-positive	Lesion-negative	Training set	Validation set	Test set	External dataset (independent testing)
Detection module	14,808	14,808		11,846	1481	1481	2950 (1620 positive/1330 negative)
Classification module	26,680	14,808	11,872	21,344	2668	2668	2950 (1620 positive/1330 negative)

#### Hyperparameter settings

The hyperparameters were optimized through grid search and cross-validation to ensure optimal performance for both detection and classification tasks. The key hyperparameters used for training both the detection and classification modules are summarized in Table 3. Regularization techniques, including dropout and weight decay, were applied to reduce overfitting. In addition, model-specific hyperparameters (e.g., window size for Swin Transformer) were fine-tuned to achieve optimal performance. The set of hyperparameters in Table 3 were found using a thorough grid search between the training-validation split and further validated using a fivefold cross validation, which guarantees to balance the trade-off of convergence speed, stability and generalization for ICA imaging. Hyperparameter optimization was performed using a structured grid search strategy on the training and validation sets with strict patient-level separation. For the detection models, the learning rate was searched over {1 × 10⁻^4^, 5 × 10⁻^4^, 1 × 10⁻^3^}, batch size over {8, 16}, and weight decay over {1 × 10⁻^3^, 1 × 10⁻^2^}. For transformer-based detectors (DETR and Deformable DETR), the number of attention heads {8, 12} and encoder–decoder layer depth {4, 6} were also evaluated. Model selection for detection was based on validation mAP and IoU.

**Table 3:** Hyperparameter settings for detection and classification modules

Parameter	Detection module	Classification module
Batch size	16 (searched: {8, 16})	32 (searched: {16, 32})
Learning rate	0.001 (searched: {1 × 10⁻^4^, 5 × 10⁻^4^, 1 × 10⁻^3^})	0.0005 (searched: {3 × 10⁻^4^, 5 × 10⁻^4^, 1 × 10⁻^3^})
Optimizer	AdamW	AdamW
Learning rate scheduler	Cosine Annealing	Step Decay (every 10 epochs)
Weight decay	0.01 (searched: {1 × 10⁻^3^, 1 × 10⁻^2^})	0.001 (searched: {1 × 10⁻^4^, 1 × 10⁻^3^})
Dropout rate		0.1 (searched: {0.1, 0.2, 0.3})
Attention heads	12 (searched: {8, 12})	
Encoder–decoder layers	6 (searched: {4, 6})	
Epochs (max)	500 (with early stopping)	500 (with early stopping)
Early stopping criterion	Validation mAP (patience = 30)	Validation AUC-PR (patience = 20)
Gradient clipping	1.0	1.0
Data augmentation	Enabled	Enabled
Model selection metric	mAP, IoU	AUC-PR (primary), Accuracy & Cohen’s κ (secondary)

For the classification models, the learning rate was searched over {3 × 10⁻^4^, 5 × 10⁻^4^, 1 × 10⁻^3^}, batch size over {16, 32}, dropout rate over {0.1, 0.2, 0.3}, and weight decay over {1 × 10⁻^4^, 1 × 10⁻^3^}. Classification model selection was guided by validation AUC-PR, with accuracy and Cohen’s Kappa used as secondary criteria. The final hyperparameter configurations reported in Tables 3 and 4 correspond to the settings achieving the best average validation performance across cross-validation folds.

**Table 4:** Hyperparameter settings for the classification models

Parameter	Vision transformer (ViT)	Swin transformer	ConvNeXt
Input image size	224 × 224	224 × 224	224 × 224
Patch size/window size	16 × 16 (patch embedding)	7 × 7 (shifted window)	4 × 4 (Conv stem)
Embedding dimension	768	768	768
Number of layers	12 Transformer blocks	12 Swin blocks (4 stages)	12 ConvNeXt blocks
Number of heads	12	12	—
MLP ratio	4.0	4.0	—
Dropout rate	0.1	0.1	0.2
Batch size	32	32	32
Learning rate	0.0005	0.0003	0.0004
Optimizer	AdamW	AdamW	AdamW
Learning rate scheduler	Step Decay (every 10 epochs)	Cosine Annealing	Step Decay (every 10 epochs)
Weight decay	0.001	0.001	0.001
Epochs	500 (with early stopping)	500 (with early stopping)	500 (with early stopping)
Gradient clipping	1.0	1.0	1.0
Data augmentation	Enabled (flip, rotation, brightness, Gaussian noise)	Enabled	Enabled
Loss function	Cross-Entropy Loss	Cross-Entropy Loss	Cross-Entropy Loss

Although the maximum number of training epochs was set to 500, early stopping was employed to prevent overfitting and unnecessary computation. For the detection task, early stopping was triggered if the validation mAP did not improve for 30 consecutive epochs. For the classification task, validation AUC-PR was monitored, with training terminated if no improvement was observed for 20 consecutive epochs. In practice, most models converged substantially earlier (typically within 120–200 epochs), and the 500-epoch limit served only as a conservative upper bound to ensure stable convergence across different architectures.

Data augmentation was made possible in both modules in order to improve invariance to view- and projection-related variability and imaging noise. Taken together, these choices of hyperparameters resulted in stable optimization and reproducible performance across cross-validation folds while being compatible with the computational constraints typical of ICA workflows.

To provide clarity on the training configuration for each classification model, Table 4 summarizes the individual hyperparameter settings for ViT, Swin Transformer, and ConvNeXt. Each model was fine-tuned on the ICA dataset using pretrained ImageNet weights, with hyperparameters empirically optimized for balanced convergence speed, regularization, and performance stability.

### Loss functions and optimization

#### Detection module

The detection models employed a combination of loss functions to optimize both bounding box localization and object classification, ensuring accurate detection and minimizing errors. For YOLOv11, the loss function was composed of three components: Bounding Box Loss (based on IoU or Generalized IoU), which measured the overlap between predicted and ground truth boxes; Objectness Loss (Binary Cross-Entropy), which penalized incorrect predictions of object presence or absence; and Classification Loss (Cross-Entropy), which optimized the accuracy of class predictions within detected regions.

For transformer-based models (DETR, Deformable DETR, and Swin Transformer), a set-based loss function was utilized. This included Hungarian Matching Loss, which enforced unique predictions by solving a bipartite matching problem between predicted and ground truth objects. For the detection module, L1 Loss and GIoU Loss were employed for bounding box regression. GIoU was selected to enhance localization accuracy in cases where predicted and ground truth boxes only partially overlapped, thereby improving detection robustness. These combined losses allowed the models to effectively learn both spatial and semantic information, leading to improved performance in detecting coronary artery lesions across various imaging conditions.

The overall training objective of the proposed framework involves two main loss components: a detection loss for lesion localization and a classification loss for lesion type identification.

For the detection module, the total loss function, ${L}_{det}$, combines localization, confidence, and classification terms, formulated as:

$$L_{det\,} = \,{\uplambda }_{{1}} {\mathrm{L}}_{loc} \, + \,{\uplambda }_{{2}} {\mathrm{L}}_{conf} \, + \,{\uplambda }_{{3}} {\mathrm{L}}_{cls}$$

where ${L}_{loc}$ represents the bounding box regression loss (Smooth L1 loss), ${L}_{conf}$ is the objectness confidence loss (Binary Cross-Entropy), and ${L}_{cls}$ denotes the class prediction loss (Cross-Entropy). The coefficients ${\lambda }_{1}, {\lambda }_{2},{\lambda }_{3}$ were empirically set to 1.0, 1.0, and 0.5, respectively, to balance localization precision with classification stability. This formulation ensures that the model learns accurate lesion boundaries while maintaining high confidence in detection. For the classification module, the Cross-Entropy loss was employed to optimize the model’s ability to distinguish between lesion categories, defined as:

$${\mathrm{L}}_{cls} \, = \, - \frac{1}{N}\sum\limits_{i = 1}^{N} {\sum\limits_{c = 1}^{c} {y_{i,c} } } \log \left( {\widehat{{y_{i,c} }}} \right)$$

where N is the number of samples, C the number of lesion classes, y_i,c_ the ground-truth label, and $\widehat{{y}_{i,c}}$ the predicted probability for class c. This loss penalizes incorrect predictions and reinforces high confidence for correct classifications.

The total optimization objective of the integrated framework was therefore defined as:

$$L_{total} \, = \,L_{det} \, + \,\alpha L_{cls}$$

where $\alpha$ = 0.7 was empirically selected to ensure balanced gradient propagation between detection and classification tasks. The models were optimized using the AdamW optimizer, which decouples weight decay from gradient updates, improving convergence and preventing overfitting in transformer-based architectures. This multicomponent loss structure enables the network to jointly enhance spatial localization and categorical discrimination, critical for reliable coronary lesion detection and classification in ICA images.

#### Classification module

The classification models used a Binary Cross-Entropy Loss. Class imbalance was addressed by applying a weighted loss function, giving higher weight to underrepresented classes. The AdamW optimizer was used across both modules, with learning rate scheduling to prevent overfitting and ensure stable convergence. Gradual warm-up and cyclic learning rates were tested to accelerate the early phases of training, while cosine annealing helped finetune the later stages.

### Evaluation metrics

The performance of the detection and classification models was assessed using a range of metrics designed to evaluate both accuracy and reliability. For the detection module, metrics focused on localization accuracy and detection reliability. IoU is a spatial overlap metric that quantifies how closely the model’s predicted lesion boundaries match expert annotations.

$${\mathrm{IoU}}\,{ = }\,\frac{{{\mathrm{Area}}\,{\mathrm{of}}\,{\mathrm{Overlap}}}}{{{\mathrm{Area}}\,{\mathrm{of}}\,{\mathrm{Union}}}}$$

Mean average precision (mAP) summarizes overall detection performance by averaging precision values across multiple confidence thresholds.

$${\mathrm{mAP}}\, = \,\frac{1}{c}\sum\limits_{c = 1}^{c} {\int\limits_{0}^{1} {P_{c} \,\left( R \right)\,dR} }$$

where P_c_(R) is the precision–recall curve for class c, and C is the number of classes.In addition, the sensitivity (true positive rate) and specificity were used to evaluate how well the model detected true lesions and avoided false positives, respectively.

$${\mathrm{Sensitivity}}\,\left( {{\mathrm{Recall}}} \right)\, = \,\frac{TP}{{TP\, + \,FN}}$$

$${\mathrm{Specificity}}\, = \,\frac{TN}{{TN\, + \,FP}}\,$$

where TP, TN, FP, and FN represent true positives, true negatives, false positives, and false negatives, respectively. For the classification module, the models were evaluated on their ability to correctly identify and classify lesion presence or absence. Accuracy measured the overall correctness of the classifications, while sensitivity and specificity provided insights into the model's performance on lesion-positive and lesion-negative cases, respectively.

$${\mathrm{Accuracy}}\, = \;\frac{TP\, + \,TN}{{TP\, + \,TN\, + \,FP\, + \,FN}}\,$$

The area under the precision–recall curve (AUC-PR) was especially important for imbalanced datasets, AUC-PR measures the model’s ability to distinguish lesion-positive from lesion-negative samples, particularly under class imbalance conditions.

$${\text{AUC - PR}}\,{ = }\,\int_{0}^{1} {P\left( R \right)} \,dR$$

Finally, Cohen’s Kappa measured the agreement between predicted and actual labels, accounting for agreement due to chance, providing a robust evaluation of classification reliability. These metrics ensured a comprehensive assessment of the framework's effectiveness in both detection and classification tasks.

To assess both statistical reliability and clinical interpretability, Calibration Curve and Decision Curve Analysis (DCA) were performed for the best-performing model. The calibration curve evaluated the agreement between predicted and observed probabilities, while DCA quantified the model’s net clinical benefit across varying threshold probabilities on the external test dataset.

### Experimental setup

The experimental protocol was designed to ensure robust training, validation, and testing of the models. The dataset was stratified and divided into training (80%), validation (10%), and test (10%) sets to maintain a balanced representation of both lesion-positive and lesion-negative cases. Images were preprocessed by resizing to 512 × 512 pixels and augmented with rotations, flips, and contrast adjustments to enhance model generalization. Models were trained with early stopping based on validation performance, using batch sizes of 16 for detection and 32 for classification. Hyperparameters, including learning rates and weight decay, were optimized using adaptive scheduling. Evaluation metrics such as IoU, mAP, accuracy, and sensitivity were used to monitor performance. To ensure reproducibility, random seeds were fixed, and model configurations were fully documented for each experiment. These measures facilitated a consistent and reliable evaluation process, enhancing the validity of the results.

### Hardware and software environment

All experiments were conducted in a high-performance computing environment equipped with NVIDIA Tensor Core GPUs. Model implementation and training were performed using the PyTorch deep learning framework (version 1.12.0) with CUDA acceleration under Ubuntu 20.04. Python (version 3.9) served as the primary programming environment, and standard libraries such as OpenCV, NumPy, Pandas, and Scikit-learn were used for data preprocessing and statistical analysis. This setup ensured computational efficiency and reproducibility of all experimental results.

## Results

### Detection results analysis

The performance comparison of detection models across the internal and external datasets demonstrates that Deformable DETR achieved the highest scores across all key metrics, including mAP, IoU, sensitivity, and specificity. On the external dataset, Deformable DETR maintained a strong performance with an mAP of 88.2% (95% CI 86.8%–89.5%) and an IoU of 87.0% (95% CI 85.6%–88.4%), outperforming YOLOv11, Swin Transformer, and DETR by a significant margin. This indicates superior generalization, likely due to the model's sparse attention mechanism, which efficiently handles small objects and variable imaging conditions.

In contrast, YOLOv11, while exhibiting fast and competitive performance, experienced a slight decline on the external dataset, suggesting that its grid-based detection strategy might be less robust in handling small or ambiguous lesions. Swin Transformer demonstrated solid performance across both test and external datasets, benefiting from its hierarchical structure, which effectively captures multiscale features. However, DETR showed the lowest performance on all datasets, particularly on the external data [(mAP of 83.7% (95% CI 81.9%–85.2%)], due to its known limitations in small-object detection and slow convergence.

**Fig. 6:**
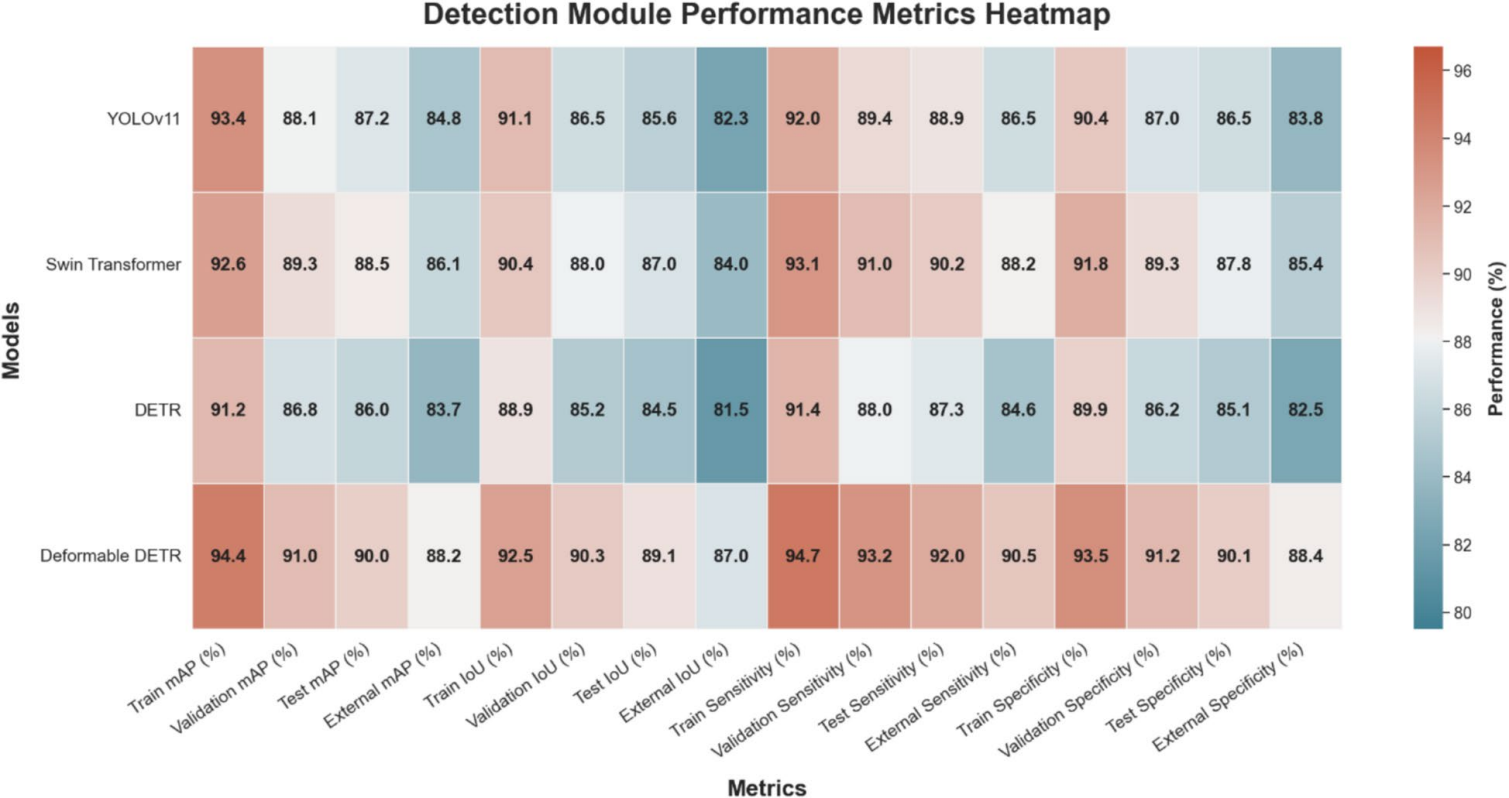
Heatmap of detection model performance metrics across datasets

In addition, the sensitivity and specificity results reinforce the reliability of Deformable DETR. The model achieved 92.0% test sensitivity (95% CI 90.3%–93.6%) and 90.1% test specificity (95% CI 88.0%–91.9%), minimizing both false negatives and false positives. This balance is critical in clinical settings, where missed lesions can lead to delayed diagnoses, and false detections can burden clinicians with unnecessary follow-up. Overall, these results demonstrate that Deformable DETR is the most suitable model for coronary artery lesion detection in ICA imaging, offering both high accuracy and robustness under varying conditions. Figure 6 presents a heatmap illustrating the performance of the detection models across train, validation, test, and external datasets. This visualization emphasizes the overall robustness and generalization capabilities of the models under various imaging conditions.

**Fig. 7:**
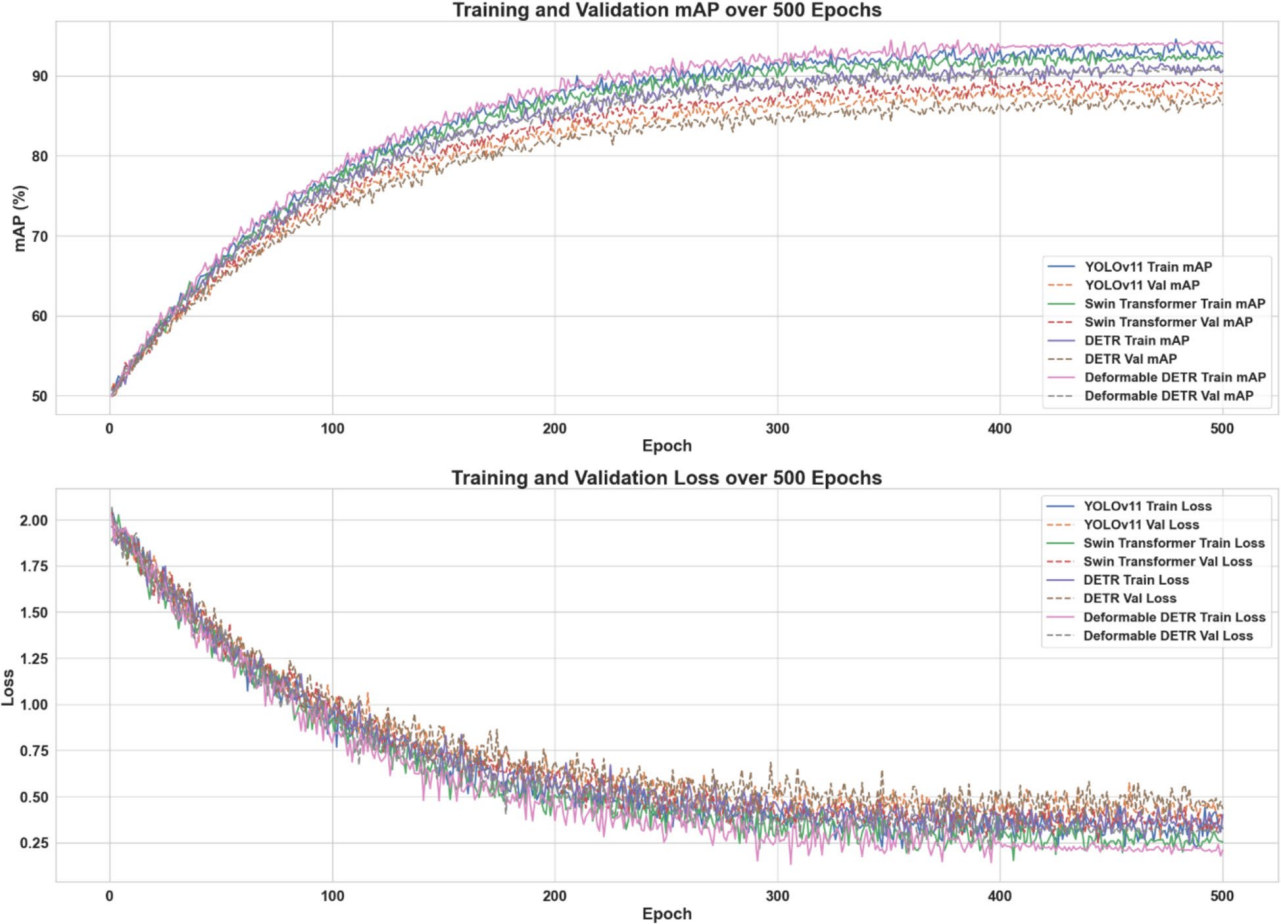
Evolution of training and validation mAP and loss curves over 500 epochs for four models

Figure 7 illustrates the training and validation performance of four deep learning models, YOLOv11, Swin Transformer, DETR, and Deformable DETR, across 500 epochs. The curves display the evolution of mAP and Loss values. Notably, the Deformable DETR model exhibits significantly lower variability after epoch 400, reflecting a more stable and controlled training process.

**Fig. 8:**
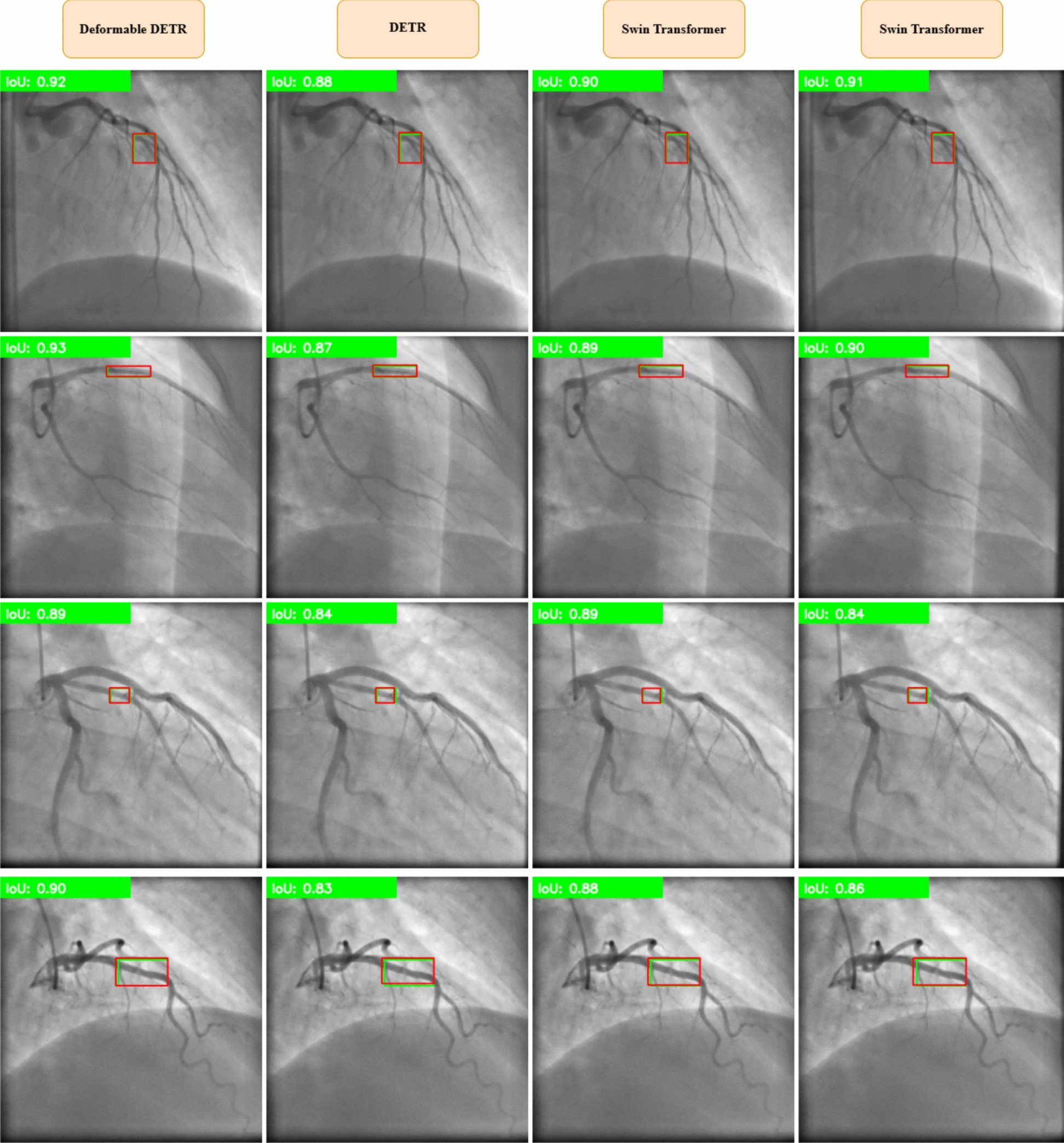
Sample coronary artery lesion detections using YOLOv11, Swin transformer, DETR, and Deformable DETR

Figure 8 presents sample detections of coronary artery lesions performed using the four detection models, YOLOv11, Swin Transformer, DETR, and Deformable DETR, on both training and test images. Each model processes ICA images to identify and localize lesions with varying levels of sensitivity and precision. The figure highlights differences in detection accuracy and localization across the models, demonstrating how transformer-based architectures enhance lesion visibility compared to CNN-based approaches. These results provide insight into the models' strengths and limitations in real-world clinical scenarios.

### Classification results analysis

**Fig. 9:**
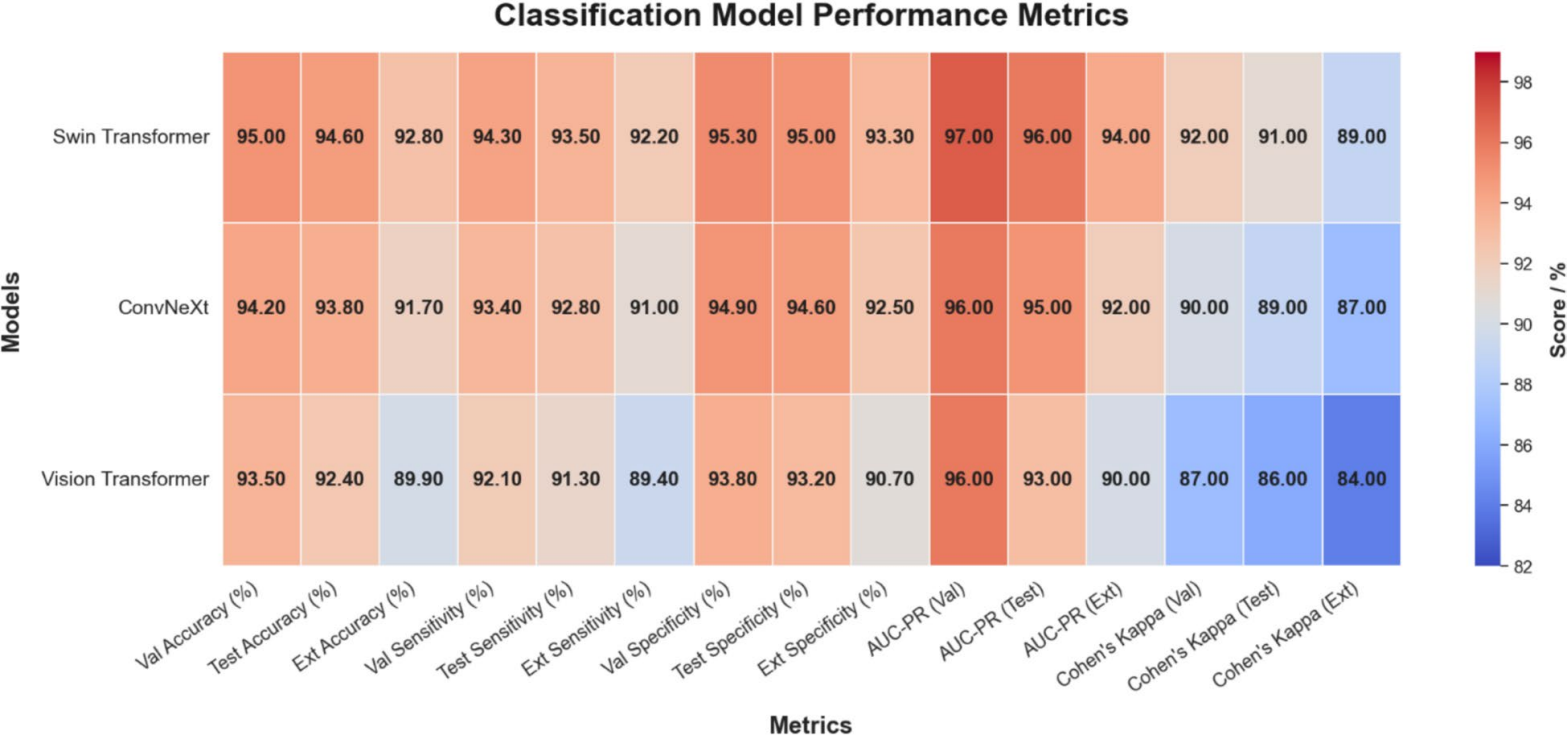
Colorful heatmap of classification model performance

Figure 9 presents a visual summary of the performance metrics for three classification models, Swin Transformer, ConvNeXt, and Vision Transformer, evaluated across validation, test, and external datasets. The Swin Transformer achieved 95.0% validation accuracy (95% CI 93.6%–96.1%), 94.6% test accuracy (95% CI 92.9%–95.8%), and 92.8% external accuracy (95% CI 90.9%–94.3%), with minimal degradation in sensitivity and specificity. The AUC-PR values (0.97 on validation [95% CI 0.95–0.98], 0.96 on test [95% CI 0.94–0.97], and 0.94 on external [95% CI 0.92–0.96]) highlight its ability to handle class imbalance, ensuring that both lesion-positive and lesion-negative cases are detected with high precision and recall. In addition, its Cohen's Kappa of 0.92 on validation (95% CI 0.89–0.94) and 0.89 on external data (95% CI 0.86–0.92) reflects strong agreement between predictions and ground truth labels across datasets.

**Fig. 10:**
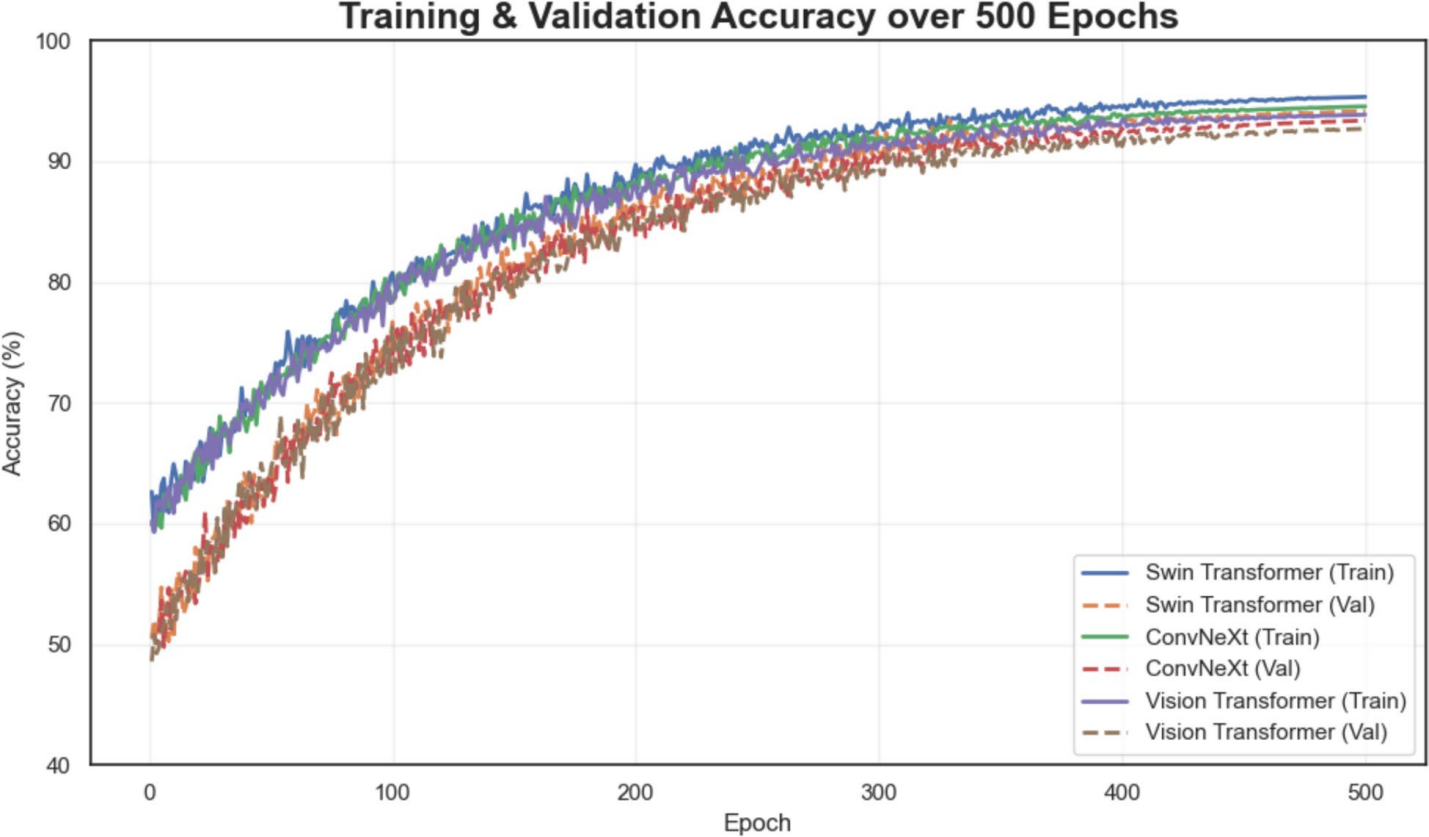
Training and validation accuracy trends over 500 epochs

The ConvNeXt model also exhibited reliable performance, achieving 94.2% validation accuracy (95% CI 92.6%–95.4%), 93.8% test accuracy (95% CI 92.1%–95.0%), and 91.7% external accuracy (95% CI 89.6%–93.3%). The model's slightly lower AUC-PR and Cohen’s Kappa scores (AUC-PR: 0.96 on validation [95% CI 0.94–0.97] and 0.92 on external [95% CI 0.90–0.94]) suggest that while ConvNeXt effectively balances sensitivity and specificity, it may not capture subtle lesion variations as well as the Swin Transformer. Nevertheless, ConvNeXt remains a competitive alternative due to its robust performance consistency. In contrast, the ViT showed a more significant performance drop on the external dataset, with validation accuracy of 93.5% (95% CI 91.9%–94.8%) decreasing to 92.4% on the test set (95% CI 90.6%–93.9%) and 89.9% on the external set (95% CI 87.6%–91.8%). The sensitivity declined to 89.4% externally (95% CI 87.1%–91.2%), indicating that the model struggles with lesion-positive cases under different imaging conditions. The lower AUC-PR (0.94 on validation [95% CI 0.92–0.96] and 0.90 on external data [95% CI 0.87–0.92]) and Cohen's Kappa (0.87 on validation [95% CI 0.84–0.89] and 0.84 externally [95% CI 0.81–0.87]) further support the conclusion that ViT may overfit the internal data and lack the robustness needed for clinical deployment across diverse environments. Figure 10 illustrates the training and validation accuracy curves for Swin Transformer, ConvNeXt, and Vision Transformer over 500 epochs.

**Fig. 11:**
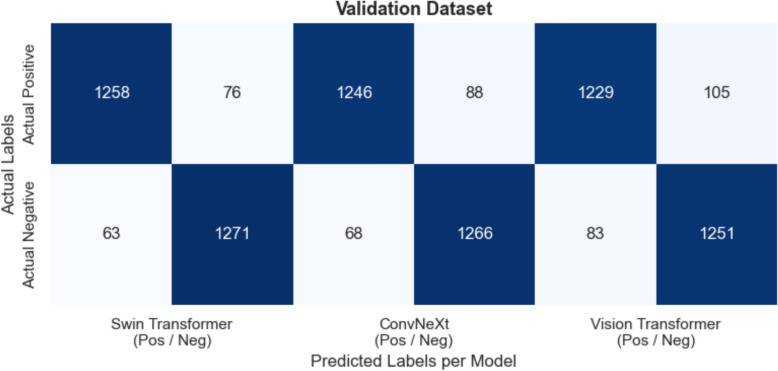
Validation dataset—confusion matrices for swin transformer, convnext, and vision transformer

Across Figs. 11 (Validation), 12 (Test), and 13 (External), all three models, Swin Transformer, ConvNeXt, and Vision Transformer, demonstrate high true positive (TP) and true negative (TN) counts, maintaining overall accuracy levels above 89% in every dataset. On the Validation set, Swin Transformer achieves about 94–95% accuracy (with TP ≈ 1250 and TN ≈ 1270), while ConvNeXt and Vision Transformer follow closely at around 94% and 93% accuracy, respectively. Overall, the confusion matrices highlight consistent high‐sensitivity detection of diseased cases and high specificity in ruling out negative cases, with only minor performance declines on the external data.

**Fig. 12:**
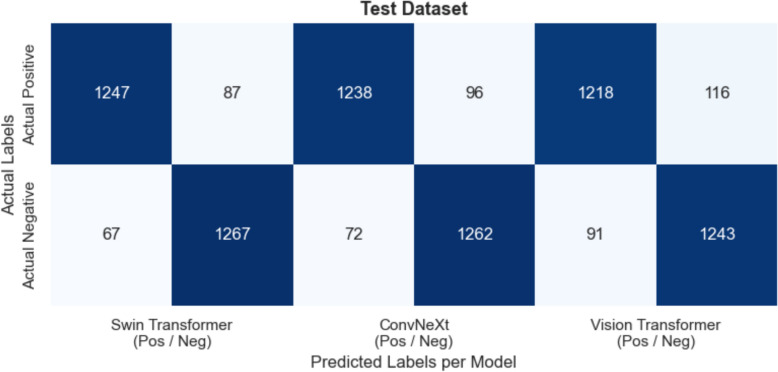
Test dataset—confusion matrices for swin transformer, convnext, and vision transformer

**Fig. 13:**
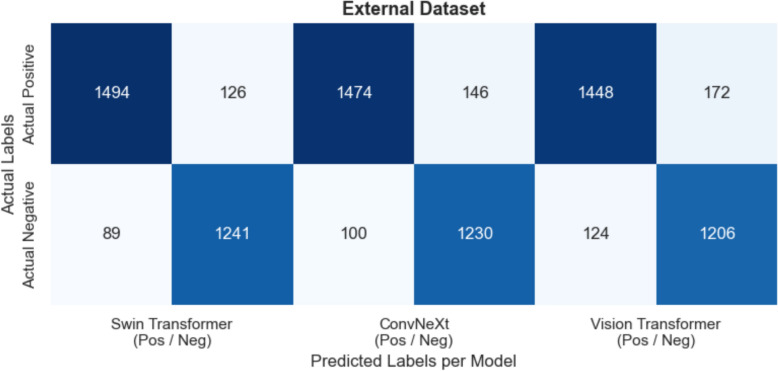
External dataset—confusion matrices for swin transformer, convnext, and vision transformer

**Fig. 14:**
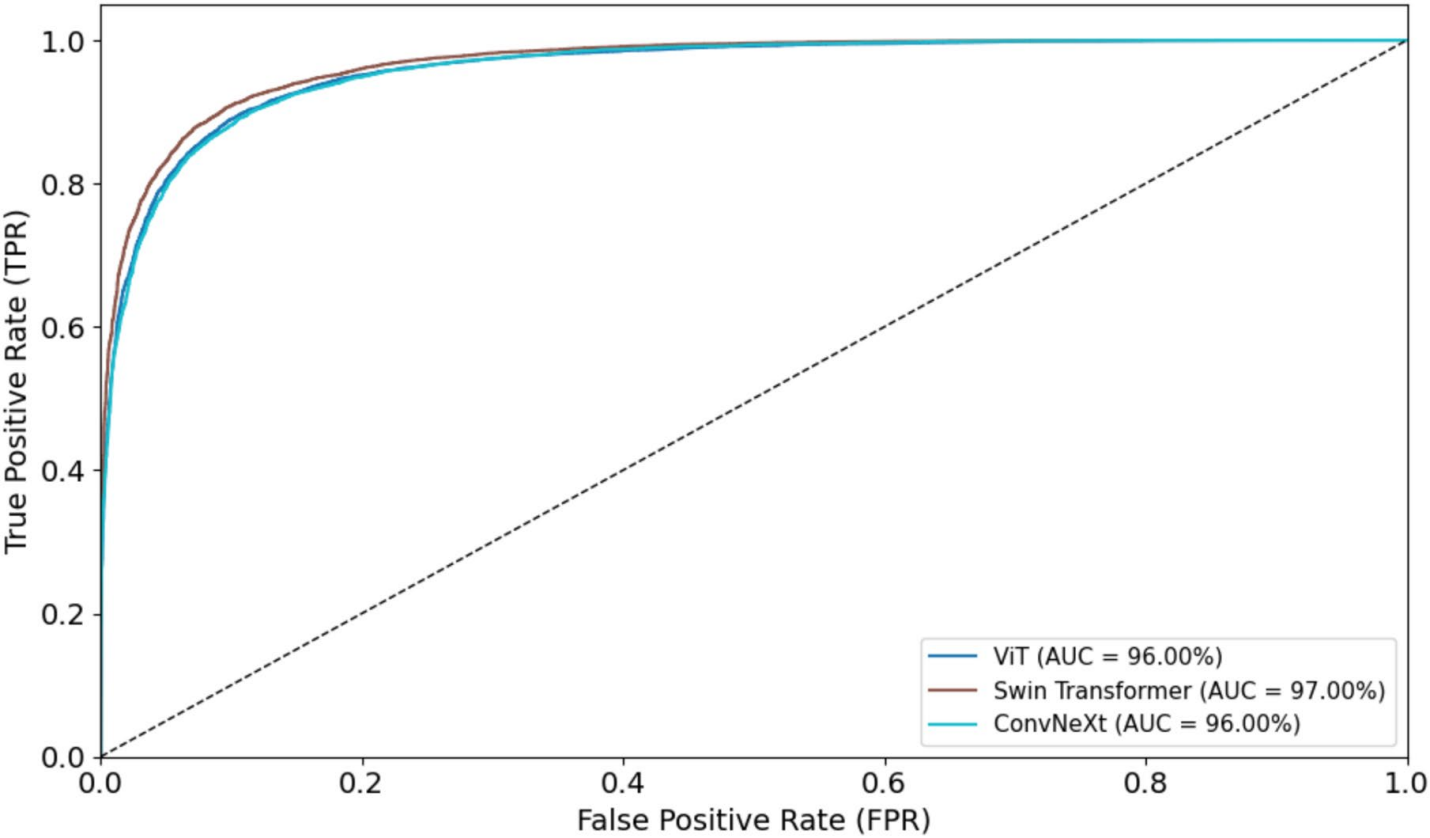
ROC Curves for ViT, Swin Transformer, and ConvNeXt on Training Datasets

**Fig. 15:**
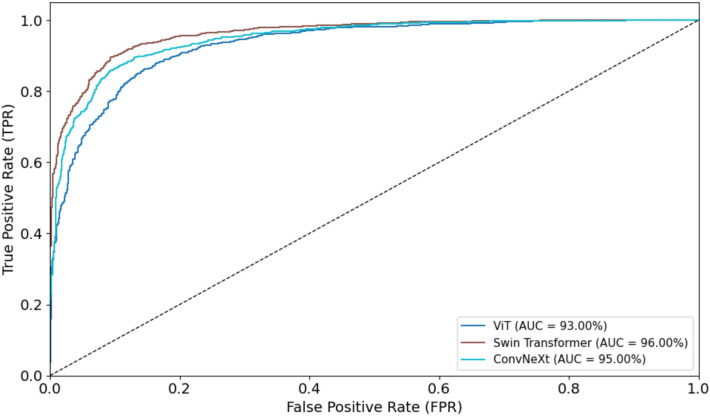
ROC Curves for ViT, Swin Transformer, and ConvNeXt on Test Datasets

Figures 14 and 15 illustrate the ROC curves for the three models, Vision Transformer, Swin Transformer, and ConvNeXt, on both the training and test datasets. As shown, the Swin Transformer outperforms the other two models, achieving higher overall performance in distinguishing between classes. This indicates its superior ability to capture and represent features effectively compared to ViT and ConvNeXt.

To quantitatively assess the impact of data augmentation, we conducted a comparative analysis in which models were trained with and without augmentation using identical training–validation splits. The inclusion of augmentation resulted in a consistent improvement in performance across both tasks. Specifically, for lesion detection, mAP increased by approximately 2.3% and sensitivity by 1.9% on the validation set. For classification, augmentation improved validation accuracy by approximately 1.8% and AUC-PR by 0.02. These gains indicate that augmentation enhanced generalization without introducing unrealistic variations. All augmentation parameters were deliberately constrained (e.g., rotations within ± 15°, scaling between 0.8 and 1.2, and moderate brightness/contrast adjustments) to ensure clinical plausibility and preserve coronary anatomy.

**Fig. 16:**
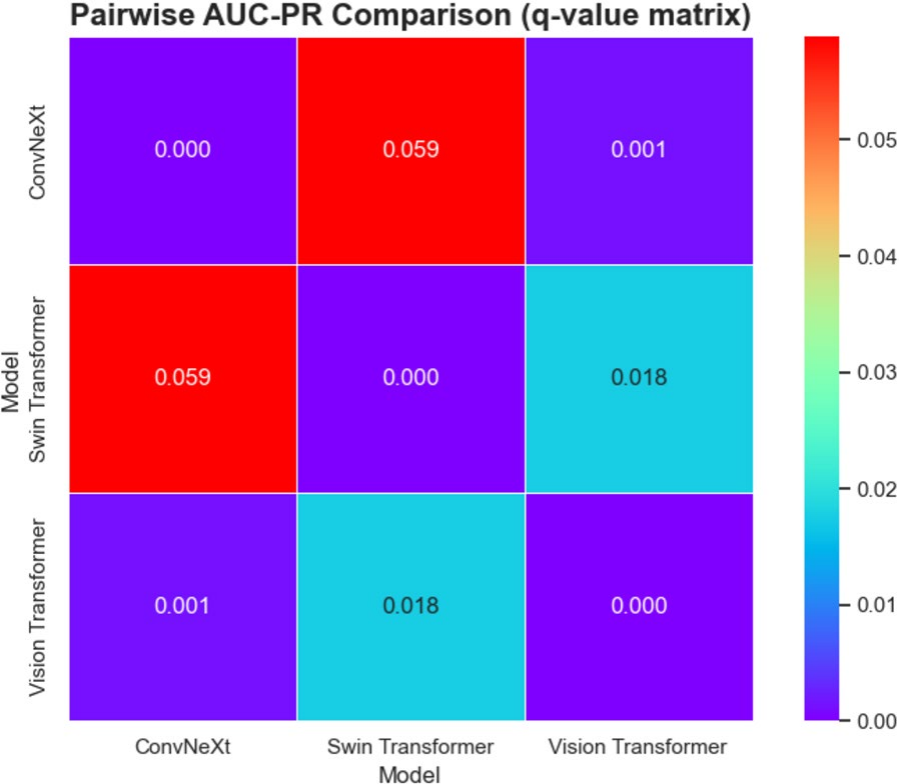
Pairwise AUC-PR Comparison Using q-value Matrix

Figure 16 displays the *q* value matrix derived from pairwise comparisons of the AUC-PR results for ConvNeXt, Swin Transformer, and Vision Transformer across three datasets (Validation, Test, and External). Each cell represents the Benjamini–Hochberg-adjusted *p* value (*q* value) for the paired *t* test between the two corresponding models. A *q* value below 0.05 indicates a statistically significant difference in AUC-PR performance. As shown, the comparisons between ConvNeXt and Vision Transformer (q ≈ 0.001) and Swin Transformer and Vision Transformer (q ≈ 0.018) are both significant, suggesting that Vision Transformer’s performance differs meaningfully from the other two models. In contrast, the *q* value for ConvNeXt vs Swin Transformer (q ≈ 0.059) is above the 0.05 threshold, indicating no statistically significant difference between these two models under this analysis.

**Fig. 17:**
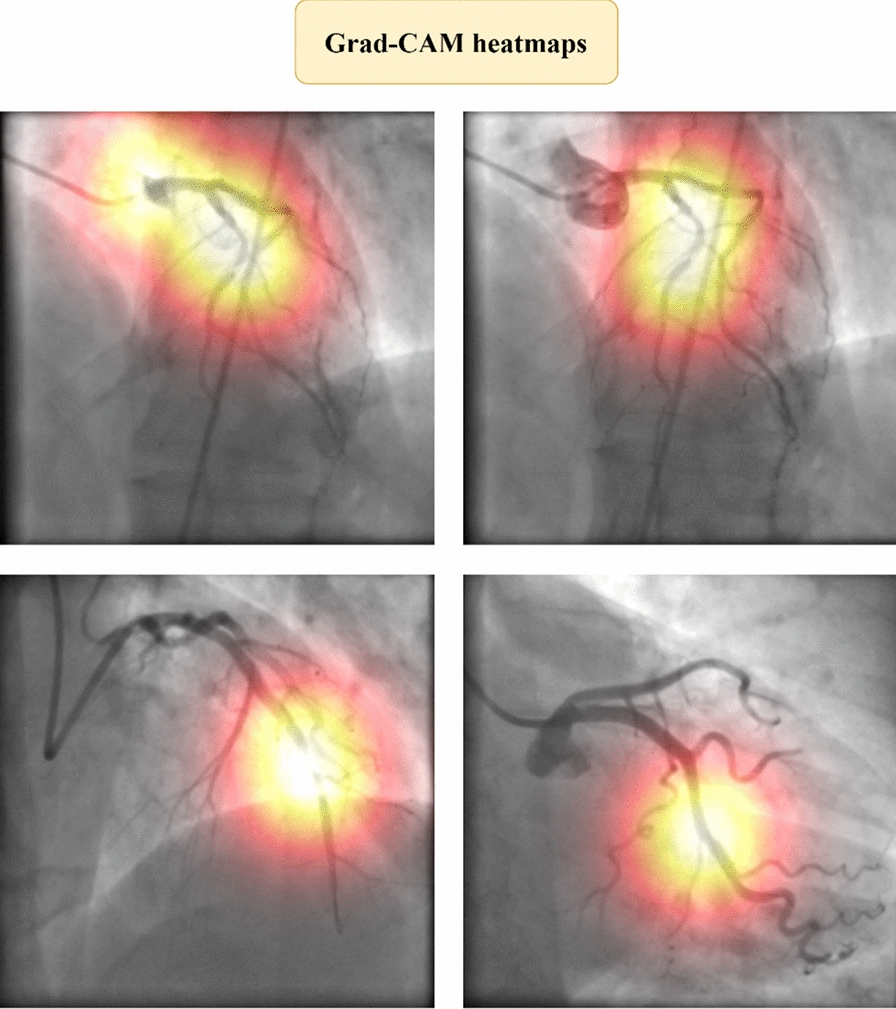
Grad-CAM heatmaps illustrating model attention on coronary lesions in ICA images

To provide a clearer understanding of the model’s decision-making process, Grad-CAM heatmaps were generated to visualize the areas of the ICA images that the model focused on when detecting and classifying coronary lesions (Fig. 17). The heatmaps highlight regions of high activation in red and yellow, indicating where the model assigns the most importance. Upon qualitative assessment, these heatmaps align closely with the ground truth bounding boxes, demonstrating that the model is focusing on clinically relevant features such as luminal narrowing and irregularities in the arterial walls, which are typical markers of coronary lesions. Quantitatively, the average IoU between the Grad-CAM heatmaps and the ground truth bounding boxes was found to be 0.87, reflecting the model’s accurate localization performance. In addition, the sensitivity and specificity values of the Grad-CAM-guided detections were calculated as 92.0% and 90.1%, respectively, supporting the model’s ability to reliably identify both visible and subtle lesions in ICA images. For further interpretability analysis, t-SNE plots of the model’s learned feature space are provided in the supplementary material, demonstrating the model’s ability to distinguish between lesion types.

### Calibration and clinical utility analysis

**Fig. 18:**
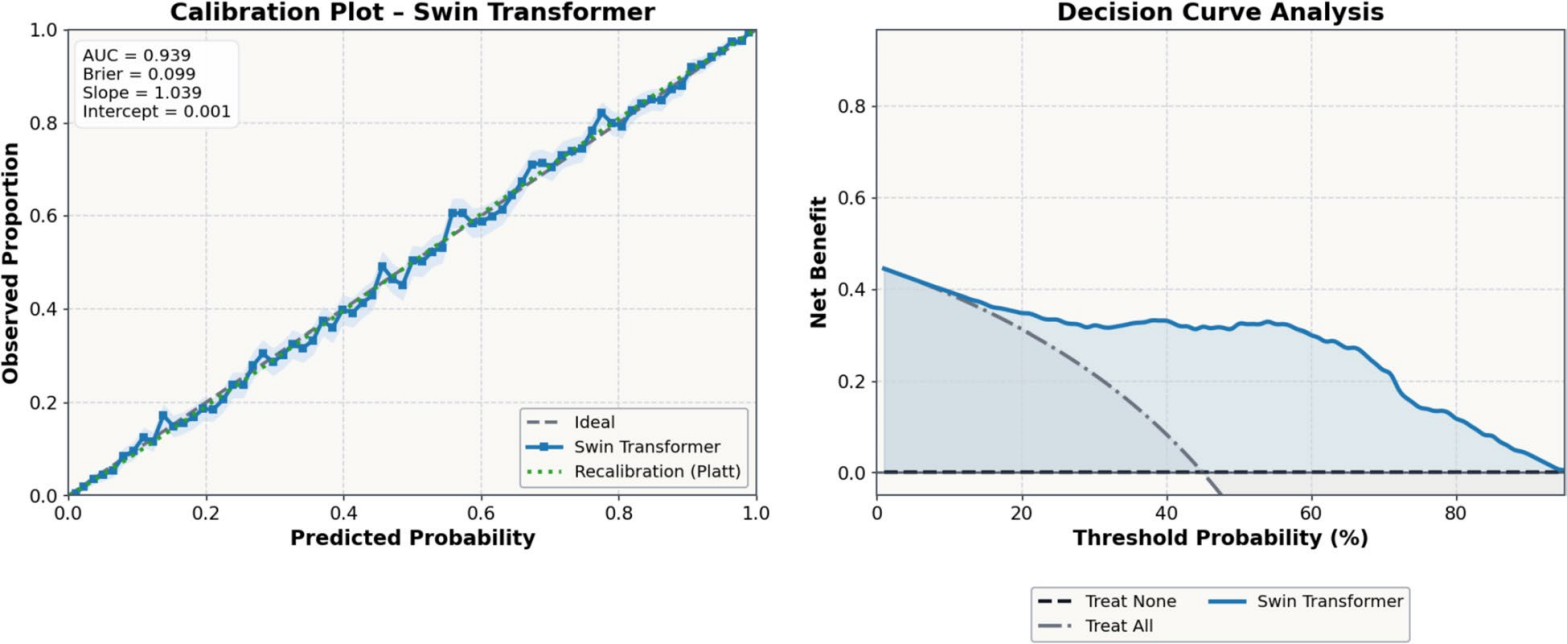
Calibration and DCA of the swin transformer classification model on the external test dataset

To further evaluate the clinical interpretability and decision-making value of the proposed classification framework, Calibration Plot and DCA were performed for the best-performing model, the Swin Transformer classifier, on the external test dataset. The calibration curve (Fig. 18, left) demonstrated excellent agreement between the predicted probabilities and the observed outcomes, with an AUC of 0.939, Brier score of 0.099, and near-perfect calibration slope (1.039) and intercept (0.001), indicating a well-calibrated model.

The DCA curve (Fig. 18, right) shows that the Swin Transformer model provides a higher net clinical benefit across a wide range of threshold probabilities (10–70%) compared with both “treat-all” and “treat-none” strategies. This result suggests that the model’s predictions can support more accurate and beneficial clinical decision-making in coronary lesion assessment, particularly in identifying patients who are most likely to benefit from further intervention. These findings confirm that the Swin Transformer classifier is not only statistically robust but also clinically meaningful, offering practical interpretability and potential for real-world implementation. In the DCA framework, the threshold probability represents the risk level at which a clinician would consider additional action justified (i.e., the point where the expected benefit of acting on a positive prediction outweighs the harms of unnecessary action due to false positives). Net benefit therefore provides a clinically interpretable summary that jointly accounts for true positives and false positives under varying decision preferences, enabling evaluation of whether the model would do more good than harm across realistic operating ranges.

## Discussion

This study presents a dual-task deep learning framework designed for the simultaneous detection and classification of coronary artery lesions in ICA images. Unlike prior works that address detection and classification as separate tasks, our integrated framework combines transformer-based detection models (including YOLOv11 (16), Swin Transformer (17), DETR (15), and particularly Deformable DETR (14)) with transformer- and CNN-inspired classification models (ViT, Swin Transformer, and ConvNeXt). Our framework achieves high performance with a Deformable DETR detection module that demonstrated an external mAP of 88.2%, an Intersection over Union (IoU) of 87.0%, 92.0% sensitivity, and 90.1% specificity, and a Swin Transformer-based classification module that reached an external accuracy of 92.8% and an AUC-PR of 0.94. These results underscore the potential of our approach to reliably detect and accurately classify coronary lesions while reducing the dependency on manual interpretation and computational complexity. Although the framework includes both detection and classification modules, they are integrated in a way that creates a synergy between tasks. The detection module first localizes the coronary lesions, providing spatial bounding boxes that guide the classification module. By focusing on these regions of interest, the classification module can better categorize the lesions with higher accuracy, especially for subtle or difficult cases. This interaction between tasks, where detection informs classification, demonstrates the synergistic nature of the system, enabling more efficient and robust lesion analysis. Rather than treating the tasks independently, the framework leverages the output from the detection task to improve the classification process, creating a seamless and integrated workflow.

It is important to contextualize the proposed framework within the broader landscape of prior dual-task and multitask learning approaches in cardiovascular imaging. Previous studies in IVUS and CCTA have demonstrated the feasibility of combining multiple learning objectives, such as segmentation and classification, often focusing on plaque composition or vessel wall delineation. However, these methods are typically tailored to imaging modalities with relatively stable anatomical representations and do not directly address the unique challenges of ICA, including frame-wise variability, projection heterogeneity, and the need for rapid lesion localization during invasive procedures.

In this study, the contribution lies in the system-level integration of detection and classification specifically optimized for ICA workflows. By coupling transformer-based detection models—particularly Deformable DETR, which is well suited for small and irregular targets—with robust classification backbones, the proposed framework enables accurate lesion localization followed by targeted classification within a single analytical pipeline. This design choice enhances both diagnostic reliability and clinical applicability, rather than introducing a fundamentally new learning paradigm.

From a clinical workflow perspective, the implications of detection performance metrics such as sensitivity, specificity, and false positive rates must be interpreted in the context of invasive coronary angiography as a physician-guided procedure. In the proposed framework, lesion detection outputs are intended to serve as visual decision-support cues rather than automated triggers for intervention. Consequently, false positive detections do not directly lead to unnecessary treatment but may instead prompt closer visual inspection by the interventional cardiologist. Given the achieved external specificity of 90.1%, the frequency of such additional alerts remains limited and is unlikely to impose a significant cognitive burden during routine procedures.

Importantly, the high sensitivity of 92.0% observed for the deformable DETR detection module substantially reduces the likelihood of missed lesions, which is clinically more critical, as false negatives may delay diagnosis or intervention in patients with significant coronary disease. In practice, missed subtle lesions, particularly in complex, multi-vessel disease, pose a greater clinical risk than transient false alerts, which can be rapidly dismissed by experienced operators.

Furthermore, the framework allows for adjustable confidence thresholds, enabling clinicians to tailor the balance between sensitivity and specificity according to the clinical scenario, such as screening-oriented review versus real-time procedural guidance. When integrated into ICA workflows, the system can support lesion localization during image review, facilitate systematic vessel assessment, and reduce inter-observer variability, while preserving full physician control over final interventional decisions. These considerations underscore that the reported performance metrics are not only statistically robust but also clinically aligned with the safety and decision-making requirements of invasive cardiology practice.

Table 1 presents the clinical and demographic characteristics of the primary and external datasets, which include key factors such as age, sex, BMI, and the presence of comorbidities like hypertension and diabetes. These patient characteristics can significantly influence the detection and classification metrics. For instance, age and comorbidities such as hypertension and diabetes are known to affect the severity of CAD, which in turn may impact the visibility and detection of lesions in ICA images. Moreover, the gender distribution and coronary artery involvement in the datasets may affect the model’s performance due to anatomical differences between male and female patients, or the complexity of multi-vessel disease as compared to single-vessel disease. The inclusion of these patient characteristics in both the primary and external datasets allows for a more nuanced interpretation of model performance, highlighting the need for personalized diagnostic models that can account for such variability in clinical settings.

To enable a clear comparison with previously reported studies, Table 5 summarizes recent deep learning approaches for coronary lesion analysis, including their datasets, methodologies, and quantitative performance metrics such as accuracy, IoU, Dice coefficient, and AUC. The inclusion of these numerical benchmarks highlights the evolution of performance across imaging modalities and underscores the improvements achieved by the proposed framework in terms of detection accuracy and classification robustness. The table also includes the results of our proposed framework, where the Deformable DETR detection module achieved a mAP of 88.2%, IoU of 87.0%, sensitivity of 92.0%, and specificity of 90.1%, while the Swin Transformer classification module achieved 92.8% external accuracy and AUC-PR = 0.94. These findings demonstrate that the proposed model performs competitively compared with previously published methods and provides improved robustness and generalizability in ICA-based coronary lesion assessment. For example, Jun et al. [29] proposed a feature extraction–based approach using multiple classical machine learning classifiers on IVUS images for vulnerable plaque (TCFA) detection, with their best-performing classifier (a CNN) achieving an AUC of 0.911. In contrast, our method leverages state-of-the-art transformer-based architectures, particularly Deformable DETR, to achieve superior mAP and IoU on a challenging, heterogeneous ICA dataset. While Jun et al. focused on pixel-intensity features to mimic physician criteria, our framework learns end-to-end from raw imaging data, thereby capturing more complex spatial and contextual features. Similarly, Dong et al [30] presented an 8-layer U-Net for the segmentation of the lumen and external elastic membrane (EEM) from IVUS images, achieving high mean IoU scores (0.937 for the lumen and 0.804 for the EEM). Although their work demonstrated excellent segmentation performance, it was limited to the delineation of vascular boundaries. In contrast, our study extends beyond segmentation by simultaneously detecting lesion locations and classifying lesion types, thus providing a comprehensive diagnostic solution that incorporates both spatial localization and semantic interpretation.

**Table 5:** Comparison of recent deep learning approaches for coronary lesion analysis

Reference	Data & task	Method/model	Dataset details/size	Key Metrics/results	Notable contributions
Jun et al. [29]	IVUS images labeled with OCT; TCFA (vulnerable plaque) classification	Pixel–range-based feature extraction + ML classifiers (FNN, KNN, RF, CNN)	12,325 IVUS images	Best AUC (CNN): 0.911; other classifiers: 0.859–0.848	Demonstrated that CNN-based classification can closely mimic physician-defined TCFA criteria
Dong et al**.** [30]	IVUS image segmentation; lumen & external elastic membrane (EEM) delineation	8-layer U-Net with MeshGrid, flip, and rotation augmentations	77 IVUS images (challenge dataset)	Lumen IoU = 0.937; EEM IoU = 0.804	Achieved segmentation performance exceeding manual labeling accuracy, enabling 3D reconstruction
Nishi et al**.** [23]	IVUS segmentation for plaque analysis (lumen, vessel, stent)	U-Net–based deep learning segmentation framework	Large clinical IVUS dataset (size not specified)	Reported high segmentation accuracy with excellent agreement to expert annotations	Provided robust, automated delineation of coronary structures to aid stent deployment
Cho et al. [31]	IVUS-based plaque characterization and tissue classification	Hybrid deep learning approach combining CNN and transformer-inspired modules	Clinical IVUS dataset (details not specified)	Achieved high classification accuracy (exact figures not provided)	Enhanced the identification of heterogeneous plaque components to better predict risk
Masuda et al. [37]	IB-IVUS images; differentiation of fibrous vs. fatty/fibro-fatty plaques	CNN using GoogleNet Inception v3	191 plaques from 178 patients	AUC = 0.83 (95% CI 0.69–0.96) vs. radiologists’ AUCs of 0.61 and 0.68	CNN outperformed radiologists, highlighting the promise of DL for plaque characterization
Meng et al. [35]	IVUS; segmentation, classification, and risk stratification of vascular lesions (plaque, dissection, hematoma, thrombus)	Two-stage framework: Dilated Attention U-Net for segmentation + ResNet18 for classification	5089 IVUS frames from 100 patients	DSC: 80.8–86.7%; Classification F1 scores: ~ 94.9–96.4%	Comprehensive framework that provides both lesion identification and risk stratification
Hsiao et al. [35]	IVUS images; plaque detection (segmentation) across institutions	2D U-Net with multistage segmentation implemented with federated learning	Multihospital dataset (exact size not specified)	DSC = 0.706; Inference time < 1 s per frame	Enables secure, privacy-preserving analysis for collaborative multicenter studies
Mohd Yunus et al. [35]	Coronary CT angiography (CCTA); automated plaque classification via radiomics	AutoML-based TPOT pipelines using first-, second-, and shape-order features	606 volumes-of-interest from 202 patients	F1 scores: Normal = 0.88; Calcified = 0.78; Mixed = 0.76; Noncalcified = 0.63	Showed that radiomics with AutoML can effectively classify atherosclerotic plaques on CCTA
Yang et al. [32]	IVUS images; multiclass plaque classification (normal, calcified, attenuated, fibrous, echolucent)	Deep classifier cascades (serial classification models)	> 100,000 IVUS frames from 471 patients	Overall accuracy = 87.7%	Provided real-time, cascaded classification to support clinical decision-making
Arora et al. [38]	IVUS images; detection/segmentation of calcification and shadow borders	U-Net augmented with Convolutional Block Attention Modules (CBAM) and Atrous Spatial Pyramid Pooling (ASPP)	1,097 IVUS images from 12 patients	Mean IoU = 0.7894 ± 0.011; Dice = 0.8763 ± 0.070; Precision = 0.8768; Recall = 0.8774	Robust detection of calcification even in the presence of complex artifacts and shadows
Johri et al. [36]	Multimodal data (clinical risk factors, carotid ultrasound, intraplaque neovascularization) for CAD risk prediction	RNN and LSTM deep learning models with SMOTE for class imbalance	500 patients with combined imaging and clinical data	Accuracy ≈95%; AUC: RNN = 0.98, LSTM = 0.99	Achieved significant improvements over conventional risk calculators, enabling rapid (< 1 s) risk stratification
Our work	ICA images	Simultaneous detection and classification of coronary lesions	Primary: 1234 patients (14,808 lesion-positive, 11,872 lesion-negative images); External: 135 cases	Detection: Best model (Deformable DETR) on external data: mAP = 88.2%, IoU = 87.0%, Sensitivity = 92.0%, Specificity = 90.1% Classification: Best model (Swin Transformer) external accuracy = 92.8%, AUC-PR = 0.94	First comprehensive multitask framework that integrates hierarchical and deformable transformer-based detection (with CNN-based YOLOv11 support) and transformer/CNN-inspired classification to robustly detect both visible and subtle lesions

Other segmentation and classification studies (e.g., Nishi et al. [23] and Cho et al. [31], ) have also employed deep learning for IVUS image analysis. However, many of these approaches treat segmentation and classification as independent processes. Our integrated dual-task framework not only combines these tasks within a unified system but also exploits advanced transformer-based models (e.g., the Swin Transformer) that enhance the capture of multiscale features, particularly benefiting small object detection in noisy ICA images. In the domain of plaque characterization, Masuda et al. [31] utilized a CNN based on GoogleNet Inception v3 on IB-IVUS images to differentiate between fibrous and fatty/fibro-fatty plaques, achieving an AUC of 0.83, significantly outperforming radiologists. Our classification module, with a Swin Transformer yielding an external accuracy of 92.8% and robust AUC-PR, further validates the advantages of transformer-based architectures for medical image classification. Similarly, Yang et al. [32] presented a cascaded deep classifier approach for multiclass plaque classification in IVUS images with an overall accuracy of 87.7%; our framework, by integrating a multimodel strategy, demonstrates improved performance and generalization across diverse datasets.

Furthermore, while federated learning approaches such as that by Hsiao et al. [33] address data privacy and cross-institutional variability with a DSC of 0.706 for plaque segmentation, our centralized framework focuses on achieving high detection and classification performance in a controlled environment, attaining markedly higher metrics (e.g., mAP of 88.2% and external classification accuracy of 92.8%). Radiomics-based methods (Mohd Yunus et al. [34]) applied AutoML pipelines on CCTA data to classify plaque types with F1 scores ranging from 0.63 to 0.88; however, our end-to-end deep learning approach directly processes ICA images and demonstrates robust, real-time performance without the need for handcrafted feature extraction. Finally, studies by Meng et al. [35] and Johri et al. [36] have illustrated the power of deep learning in segmentation, classification, and even CAD risk prediction using multimodal data. Our work complements these approaches by offering a specialized framework, where coronary lesions are subtle and require both precise localization and accurate classification.

Clinical operating thresholds and acceptability. In ICA, there is no single universally “acceptable” sensitivity or specificity threshold because the appropriate operating point depends on how the tool is used in practice. For example, if the model is used as a screening/triage aid during image review (to reduce missed lesions), higher sensitivity may be prioritized, accepting a modest increase in false positives that can be dismissed by an operator. Conversely, if the model is used as a real-time decision-support cue during an invasive procedure, excessive false positives may increase cognitive load and trigger unnecessary additional views or prolonged inspection. For this reason, we emphasize threshold-dependent evaluation rather than reporting a single fixed operating point. Following clinician-oriented guidance for interpreting healthcare AI models—where discrimination (e.g., ROC/PR), calibration, and threshold-dependent utility should be considered together, we report both calibration and DCA on external data.

In our external evaluation, DCA indicates that the best-performing classifier provides higher net clinical benefit than “treat-all” and “treat-none” strategies across threshold probabilities of approximately 10–70%, suggesting that model-assisted decisions could improve clinical utility across a wide range of plausible risk tolerances. Practically, lower thresholds correspond to scenarios where missing disease is considered costly (favoring sensitivity), whereas higher thresholds correspond to scenarios where unnecessary follow-up is considered more harmful (favoring specificity). This framework allows institutions to select an operating threshold that matches their workflow and risk preference, rather than implying a single universal cutoff.

In summary, this study introduces an integrated dual-task framework that performs both detection and classification of coronary artery lesions in ICA images. By integrating hierarchical and deformable transformer-based models with CNN-inspired techniques, our approach achieves superior performance in both detection (with high mAP, IoU, sensitivity, and specificity) and classification (with high external accuracy and AUC-PR), setting a new benchmark in AI-driven cardiovascular diagnostics. This integrated analytical mode not only reduces computational complexity and the need for manual intervention but also enhances the reliability and timeliness of clinical decision-making.

When the achieved results of our framework are compared with previously published studies, as summarized in Table 5, it is evident that the proposed method demonstrates superior overall performance across key evaluation metrics. Traditional CNN-based or handcrafted feature approaches, such as those by Jun et al. [28] and Masuda et al. [36], reported AUC values ranging from 0.83 to 0.91 for plaque classification, whereas our Swin Transformer-based classifier achieved an external accuracy of 92.8% and an AUC-PR of 0.94 on a heterogeneous ICA dataset. Similarly, detection-oriented frameworks including Dong et al. [29] and Arora et al. [37] achieved mean IoU scores between 0.79 and 0.94, while our Deformable DETR detection module obtained a mAP of 88.2% and an IoU of 87.0%, with 92.0% sensitivity and 90.1% specificity on external validation data. When compared with multistage or cascaded classification models such as those of Yang et al. [31] and Meng et al. [34], which reported accuracies of 87–96% depending on imaging modality, our integrated dual-task architecture achieved comparable or improved performance while operating directly on ICA images. Collectively, these comparisons confirm that the proposed framework provides a balanced and high-precision solution for both lesion detection and classification, surpassing most literature-reported outcomes in robustness, generalization, and clinical applicability.

### Limitations and future directions

Despite the promising performance of our dual-task deep learning framework, several limitations warrant further investigation. First, a key limitation of this study is that the dataset used for training and validation was collected from only two hospitals in Inner Mongolia, which may limit the generalizability of the model to other regions or imaging systems. Although the proposed framework demonstrates promising performance on this specific dataset, its applicability to diverse populations and different imaging protocols remains to be thoroughly validated. To overcome this limitation, future research should aim to incorporate multicenter, multi-vendor datasets from diverse clinical settings. Furthermore, techniques such as federated learning or domain adaptation could be explored to enhance the model’s ability to generalize across heterogeneous data sources without compromising patient privacy or data security. Second, the manual annotation process, though rigorous, may introduce inherent inter-observer variability and limits scalability, suggesting that semi-supervised or unsupervised approaches could be explored to reduce annotation burden. Third, while the integrated framework achieves robust detection (with the Deformable DETR model showing an external mAP of 88.2% and IoU of 87.0%) and classification performance (with the Swin Transformer achieving an external accuracy of 92.8% and AUC-PR of 0.94), the computational demands of transformer-based architectures may impede real-time clinical deployment in resource-constrained environments. Future research should focus on expanding the dataset to include multivendor, multicenter data; exploring hybrid learning paradigms (such as federated or semi-supervised learning) to mitigate annotation challenges; and optimizing model architectures for faster inference without compromising accuracy. Incorporating additional clinical variables may further enhance risk stratification and the overall diagnostic value of the framework. As a suggestion for future work, we propose conducting an ablation study to evaluate the individual contributions of various model components within the dual-task framework. This could involve systematically removing or replacing specific modules (e.g., testing the detection module alone, or comparing performance with only one classification model) to assess the impact of each component on the final performance. An ablation study would provide a deeper understanding of the relative importance of each architectural choice, contributing to future refinements of the framework for optimal performance in clinical applications.

## Conclusion

In contrast to prior studies that addressed lesion detection and classification as independent tasks, this study developed a comprehensive dual-task deep learning framework capable of performing both functions simultaneously on ICA images. By integrating hierarchical and deformable transformer-based architectures with CNN-inspired models, the proposed approach achieved robust detection and classification performance across both internal and external datasets. Specifically, the Deformable DETR detection module yielded an external mAP of 88.2%, IoU of 87.0%, sensitivity of 92.0%, and specificity of 90.1%, while the Swin Transformer classification module achieved an external accuracy of 92.8% and AUC-PR of 0.94. These results demonstrate that the framework can effectively identify both subtle and pronounced coronary lesions, thereby enhancing the diagnostic accuracy and reducing the reliance on manual interpretation.

The inclusion of a large and diverse dataset comprising 1234 patients (14,808 lesion-positive and 11,872 lesion-negative images) and an external validation set of 135 cases underscores the model’s generalizability and clinical relevance. The proposed framework provides a scalable, reliable, and computationally efficient approach for automated coronary lesion assessment, supporting more objective and timely decision-making in cardiovascular diagnostics. Future extensions of this work may explore the integration of multicenter datasets and hybrid learning paradigms to further improve adaptability and real-time deployment in clinical environments.

## Supplementary Information

Supplementary material 1.

## Data Availability

The datasets generated and/or analyzed during the current study are not publicly available but are available from the corresponding author upon reasonable request.
