# Supplementary material for: A dual-task deep learning framework for automated detection and classification of coronary artery lesions in invasive coronary angiography imaging
Source: Eur J Med Res. 2026 Jan 30;31:352. doi: 10.1186/s40001-025-03818-3 (PMC12930670; doi:10.1186/s40001-025-03818-3)
Supplement: Supplementary file 1 — Supplementary material 1. [file 40001_2025_3818_MOESM1_ESM.docx]

Figures S1, S2, and S3 illustrate the t-SNE embeddings of the Validation, Test, and External datasets for Swin Transformer, ConvNeXt, and Vision Transformer, respectively. Each figure consists of three rows (one per dataset) and two columns: "Before t-SNE" (left) and "After t-SNE" (right). The "Before t-SNE" plots remain identical across models within each dataset, as they reflect the same original feature space before being processed by the models. The "After t-SNE" plots, however, differ for each model, visualizing how their respective confusion matrices influence the final separability of positive (lesion) and negative (non-lesion) cases. The visualization highlights how each model learns different decision boundaries, with Swin Transformer achieving the most distinct class separation, followed by ConvNeXt and Vision Transformer, which exhibit slightly more overlap in the embedded space, corresponding to their lower validation and test performance.


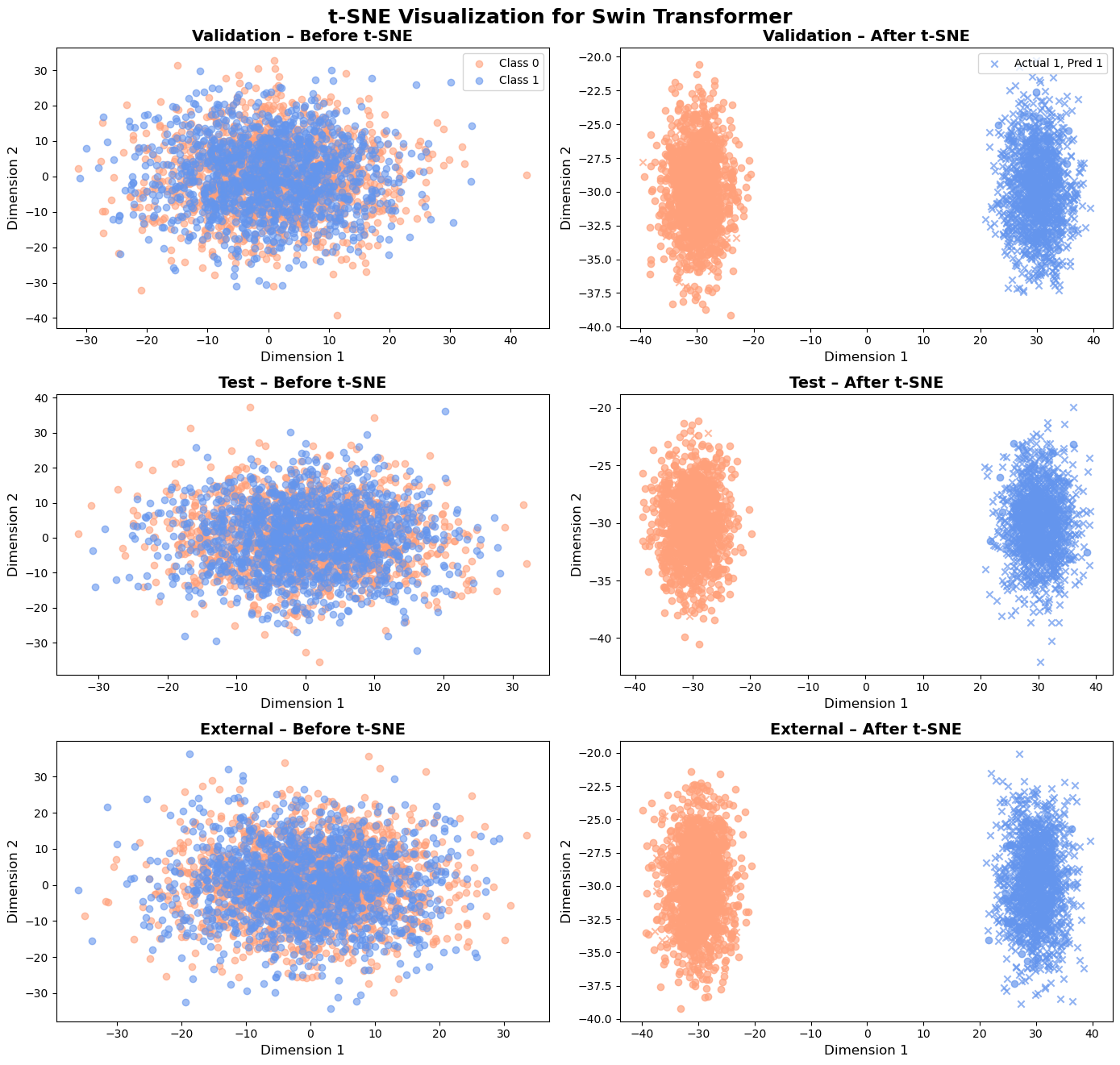


**Figure S1**.t-SNE Visualization for Swin Transformer Across Validation, Test, and External Datasets


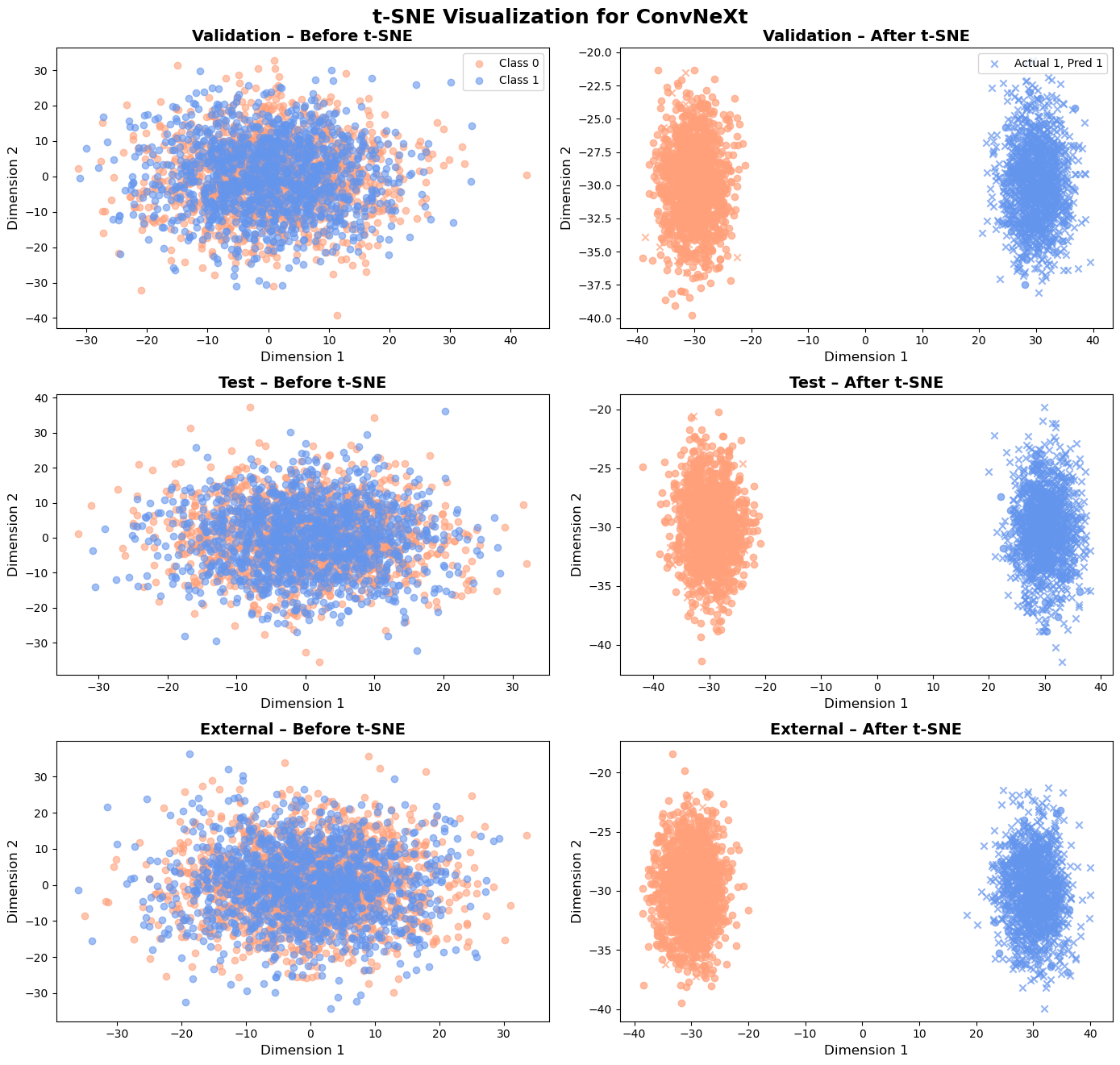
**Figure S2**. t-SNE Visualization for ConvNeXt Across Validation, Test, and External Datasets


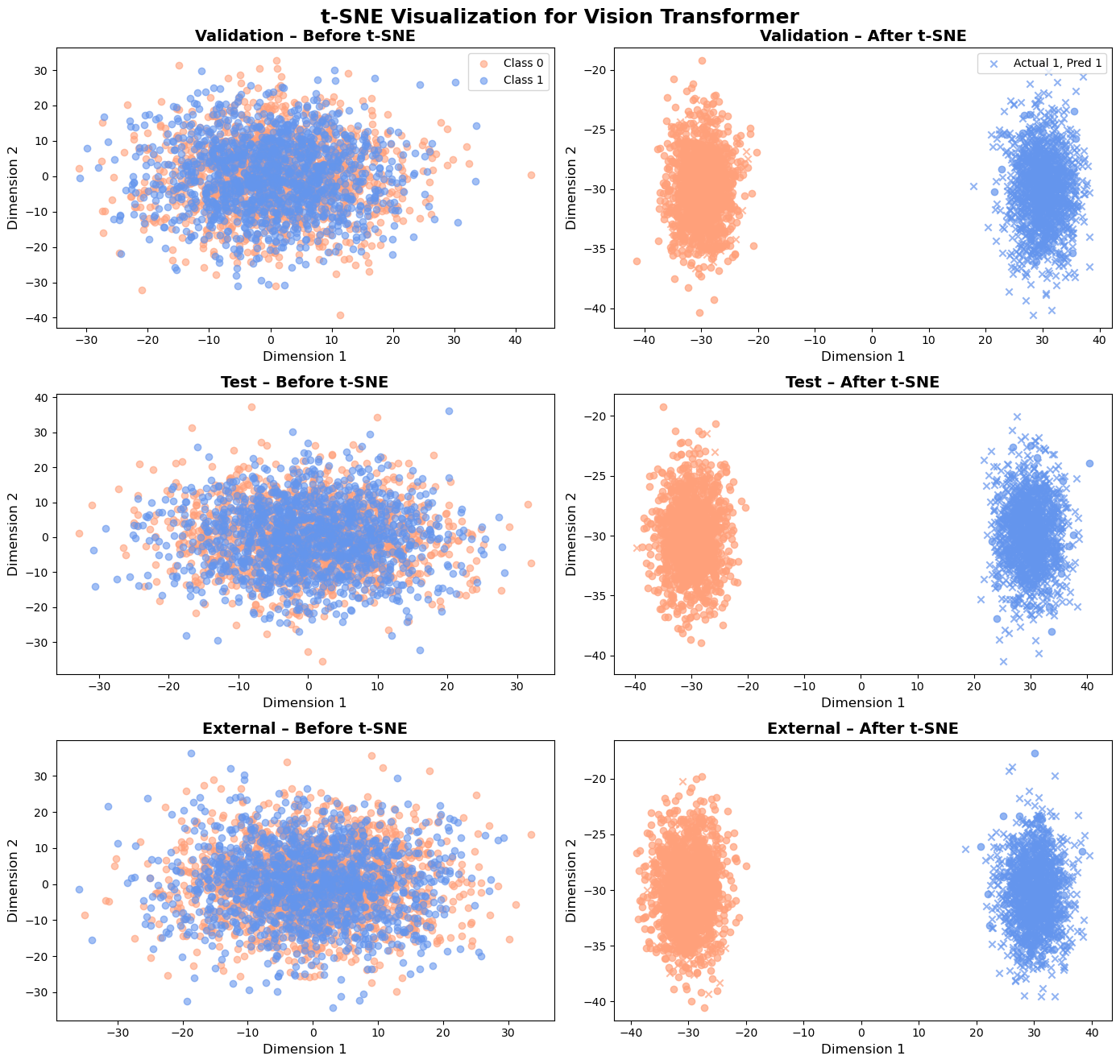
**Figure S3**. t-SNE Visualization for Vision Transformer Across Validation, Test, and External Datasets
